# Regulação assistencial e governança regional nas estratégias de microeliminação das hepatites virais

**DOI:** 10.1590/0102-311XPT104125

**Published:** 2026-06-26

**Authors:** Josué Souza Gleriano, Carlise Krein, Claudia Beatriz Cunha de Oliveira, Stella Giansante, Marcia Oliveira de Souza, Gessé Duque Ferreira de Oliveira, Regina Mary da Silva Nascimento, Lucieli Dias Pedreschi Chaves

**Affiliations:** 1 Universidade do Estado de Mato Grosso, Tangará da Serra, Brasil.; 2 Escola de Enfermagem de Ribeirão Preto, Universidade de São Paulo, Ribeirão Preto, Brasil.; 3 Secretaria Municipal de Saúde de Tangará da Serra, Tangará da Serra, Brasil.; 4 Secretaria de Estado de Saúde de Mato Grosso, Cuiabá, Brasil.

**Keywords:** Hepatite Viral Humana, Assistência Integral à Saúde, Serviços de Saúde, Sistemas de Saúde, Viral Human Hepatitis, Comprehensive Health Care, Health Services, Health Systems, Hepatitis Viral Humana, Atención Integral de Salud, Servicios de Salud, Sistemas de Salud

## Abstract

O estudo objetivou avaliar a gestão da regulação assistencial na rede de atenção à saúde, bem como o processo de elaboração e consenso de ações estratégicas pactuadas para o enfrentamento das hepatites virais, na perspectiva da governança regional. Trata-se de pesquisa-ação, de abordagem qualitativa, realizada em uma Região de Saúde de Mato Grosso, Brasil, com a participação de 45 profissionais atuantes na vigilância, cuidado, regulação, gestão e controle social. A realização de grupo focal possibilitou a análise conjunta das estratégias de enfrentamento, culminando na formulação de consenso regional e pactuação colegiada. Os resultados evidenciam, no nível político-institucional, a ausência de estratégias para enfrentar vazios assistenciais, a centralização da regulação e a fragilidade dos processos de informatização. No nível organizacional, destacam-se dificuldades de integração horizontal, com uma rede fragmentada e limitada capacidade de suporte clínico descentralizado. No nível das práticas, persistem falhas na articulação intersetorial, informalidade nos fluxos de cuidado e fragilidade da atenção primária à saúde (APS) no monitoramento pós-diagnóstico. O plano de ação consensuado contempla quatro linhas estratégicas: ampliar o acesso às ações de prevenção; fortalecer a atenção especializada articulada à APS; expandir a capacidade diagnóstica laboratorial; e consolidar a vigilância em saúde mediante pactuações interinstitucionais. A eliminação das hepatites virais demanda atuação sistêmica, orientada pela justiça social e pela construção de soluções coletivas que respondam às necessidades regionais, com monitoramento contínuo para o alcance das metas globais até 2030.

## Abstract (en)

This study aimed to evaluate the management of care regulation in the health care network and the process of elaboration and consensus of strategic actions to cope with viral hepatitis based on regional governance. This qualitative action research was carried out in a Mato Grosso Health Region, Brazil, with 45 professionals working in surveillance, care, regulation, management, and social control. The focus group enabled the joint analysis of coping strategies and the formulation of regional consensus and collegiate agreement. The results show the political-institutional absence of strategies to address care gaps, the centralization of regulation, and the fragility of computerization processes. Organizational difficulties in horizontal integration stand out, including a fragmented network and limited capacity for decentralized clinical support. Practices still show failures in intersectoral articulation, informality in care flows, and frail primary health care (PHC) in post-diagnosis monitoring. The consensual action plan included four strategic lines: expanding access to prevention actions, strengthening specialized care articulated with PHC, expanding laboratory diagnostic capacity, and consolidating health surveillance by inter-institutional agreements. Eliminating viral hepatitis requires systemic actions guided by social justice and the construction of collective solutions that meet regional needs and produce continuous monitoring to achieve the global goals by 2030.

## Abstract (es)

Este estudio tuvo como objetivo evaluar la gestión de la regulación asistencial en la red de asistencia sanitaria, así como el proceso de elaboración y consenso de acciones estratégicas consensuadas para combatir las hepatitis virales desde la perspectiva de la gobernanza regional. Se trata de una investigación-acción, con enfoque cualitativo, realizada en una Región Sanitaria de Mato Grosso, Brasil, con la participación de 45 profesionales que trabajan en vigilancia, atención, regulación, gestión y control social. El grupo focal permitió el análisis conjunto de estrategias de afrontamiento, lo cual culminó en la formulación de consenso regional y acuerdo colegiado. Los resultados muestran, a nivel político-institucional, la ausencia de estrategias para enfrentar las brechas asistenciales, la centralización de la regulación y la fragilidad de los procesos de informatización. A nivel organizacional destacan las dificultades de integración horizontal, con una red fragmentada y limitada capacidad de apoyo clínico descentralizado. A nivel de prácticas persisten fallas en la articulación intersectorial, informalidad en los flujos asistenciales y fragilidad de la atención primaria de salud (APS) en el seguimiento posdiagnóstico. El plan de acción consensuado incluye cuatro líneas estratégicas: ampliar el acceso a las acciones de prevención; fortalecer la atención especializada articulada a la APS; ampliar la capacidad de diagnóstico de laboratorio; y consolidar la vigilancia de la salud mediante acuerdos interinstitucionales. La eliminación de las hepatitis virales requiere una acción sistémica guiada por la justicia social y la construcción de soluciones colectivas que respondan a las necesidades regionales, con un monitoreo continuo para alcanzar los objetivos globales para 2030.

## Introdução

A gestão de um sistema de saúde deve ordenar recursos e ações para sustentar redes integradas, ajustando o cuidado às necessidades locais ^1^. Para os países latino-americanos, a Organização Pan-Americana da Saúde (OPAS) propôs o marco conceitual das Redes Integradas de Serviços de Saúde (RISS) com a atenção primária à saúde (APS) como ordenadora da rede de atenção. No Brasil, adota-se o conceito de Rede de Atenção à Saúde (RAS), que organiza a regionalização para a integração dos serviços no Sistema Único de Saúde (SUS) ^2^.

Nos sistemas de saúde do Canadá e da Europa, a regionalização fortaleceu autoridades sanitárias regionais ^3^. Na América Latina, a segmentação dos sistemas de saúde amplia os desafios para a organização das RISS ^4^. No Brasil, embora o SUS promova equidade ao garantir acesso universal, sua rede se caracteriza mais pela descentralização que pela regionalização efetiva ^5^ ^,^ ^6^, impondo desafios técnico-políticos à concretização de seus princípios.

A resposta dos sistemas de saúde às hepatites virais, reconhecidas como problema global de saúde pública e relevantes para a carga de cuidados ^7^ ^,^ ^8^, depende da capacidade de organizar o acesso por meio das RISS. As hepatives virais ganharam centralidade na Meta 3 da Agenda 2030 dos Objetivos do Desenvolvimento Sustentável (ODS), que destaca a urgência de ampliar prevenção e controle, sobretudo das hepatites B e C, devido aos altos custos para a saúde pública ^9^.

No Brasil, os ODS orientam o SUS no enfrentamento das hepatites virais, com foco em vigilância, prevenção, diagnóstico e tratamento. Tal perspectiva demonstra como as desigualdades sociais e territoriais influenciam a resposta dos sistemas de saúde e reforçam a necessidade de inovação, equidade e participação social. A governança regional fortalece a coordenação da RAS e a pactuação de estratégias para a microeliminação das hepatites virais ^10^. 

No SUS, a criação do Programa Nacional para a Prevenção e o Controle das Hepatites Virais (PNHV), em 2002, marcou uma inflexão ao reconhecer oficialmente as hepatites virais na agenda de saúde pública. Ações como a vacinação contra hepatite B, iniciada em 1989 para grupos específicos e ampliada em 2016, e a vacinação contra hepatite A, incorporada em 2014 ^11^, além da descentralização da testagem, expansão da rede de serviços para diagnósticos e a incorporação de antivirais de ação direta (*direct-acting antiviral* - DAA), com taxas de cura superiores a 95%, ainda não foram suficientes para assegurar o alcance das metas de eliminação ^12^. A distância entre o potencial científico e os resultados práticos demonstra barreiras que ultrapassam a dimensão clínica e exigem análise sistêmica em múltiplos níveis de gestão ^13^.

Diante desse cenário, torna-se necessário um esforço articulado entre distintos *stakeholders* para formular planos de enfrentamento que considerem os contextos locais e regionais, favorecendo a eliminação das hepatites virais. Nesse processo, é imprescindível que a gestão observe a descentralização sob a ótica da regionalização, valorizando espaços institucionais de pactuação e deliberação, especialmente na gestão colegiada, para que os planos dialoguem com as especificidades de cada região.

Compreende-se, portanto, que - neste estudo - o conceito de gestão não se refere a um cargo ou a uma pessoa, mas às interações, relações e pactuações institucionais estabelecidas por um conjunto de *stakeholders* com poder decisório sobre as agendas da política de saúde, abrangendo a formulação de propostas, normas e legislações, bem como a definição de prioridades, a condução de discussões e a operacionalização estratégica de ações técnicas, administrativas e políticas ^11^.

Com base nessas considerações, suscitam-se as seguintes questões: como se configura a atenção às hepatites virais sob a perspectiva da gestão do cuidado integral em uma rede de saúde regionalizada? E quais estratégias podem ser pactuadas no âmbito da gestão regional do SUS, respeitando-se o processo local de reconhecimento das hepatites virais como um problema de saúde pública? Nesse contexto, este estudo tem por objetivo avaliar a gestão da regulação assistencial na rede de atenção à saúde, bem como o processo de elaboração e consenso de ações estratégicas pactuadas para o enfrentamento das hepatites virais, na perspectiva da governança regional.

## Método

Trata-se de uma pesquisa-ação que integra produção de conhecimento e intervenção prática em ciclos de diagnóstico, planejamento, ação e reflexão, conduzidos coletivamente com os atores envolvidos ^14^. As hepatites virais foram eleitas como condição traçadora ^15^ para analisar a gestão do cuidado integral em uma RAS e pactuar soluções. O estudo foi realizado no Estado de Mato Grosso, Brasil, na Região de Saúde (RS) Médio Norte, composta por dez municípios, caracterizada por áreas rurais remotas, ampla cobertura de APS e centralização da referência às hepatites virais em um único Centro de Testagem e Aconselhamento/Serviço de Assistência Especializada (CTA/SAE) ^16^. No estudo, as RS são concebidas como espaços estratégicos para integração dos serviços e coordenação do cuidado, sendo orientadas por um planejamento regional que favorece a cooperação intergestora e o fortalecimento da rede de atenção ^17^.

O estudo foi desenvolvido em três etapas: (1) constituição de grupo gestor, formado por pesquisadores, gestores e profissionais dos serviços de referência, para levantamento da situação das hepatites virais e estratégias de enfrentamento por meio de análise documental e bibliográfica; (2) realização de grupos focais com *stakeholders*, a fim de compreender a dinâmica da gestão do cuidado integral em rede; e (3) plenária orientada por documento norteador, que buscou consenso entre representantes da RS para definição das linhas estratégicas e elaboração do plano de ação.

Entre março e maio de 2023, realizaram-se reuniões para analisar o contexto da RS na atenção às hepatites virais, utilizando dados do Departamento de Informação e Informática do SUS (DATASUS). Para contextualizar possíveis ações de enfrentamento das hepatites virais para a RS, foram priorizados documentos primários diretamente relacionados à organização e funcionamento da rede de atenção às hepatites virais, como documentos normativos internacionais e nacionais, estratégias de enfrentamento das hepatites virais em sistemas de saúde, recomendações da Organização Mundial da Saúde (OMS) e da OPAS, diretrizes ministeriais do PNHV e o plano nacional para eliminação das hepatites virais 2023-2030 ^18^, além de dados extraídos dos planos municipais ^19^ ^,^ ^20^ ^,^ ^21^ ^,^ ^22^ ^,^ ^23^ ^,^ ^24^ ^,^ ^25^ ^,^ ^26^ ^,^ ^27^ ^,^ ^28^ e Estadual de Saúde ^29^. 

Para garantir a relevância e a atualização do material, realizou-se uma revisão bibliográfica narrativa em bases como LILACS, PubMed e Embase, com foco em estratégias de microeliminação de hepatites virais, implementada por meio da estratégia de busca (“Hepatitis B” OR “Hepatitis B Virus” OR “Hepatitis C” OR “Hepatitis C Virus” OR “Viral Hepatitis”) AND (“Microelimination” OR “Micro-elimination” OR “Microelimination Strategies”) AND (“Public Health Strategies” OR “Health Policy” OR “Health Programs” OR “Disease Eradication” OR “Disease Elimination” OR “Strategic Planning”) AND (“Primary Health Care” OR “Community Health Services” OR “Integrated Health Care” OR “Program Implementation” OR “Care Coordination”), que serviu de base conceitual para estruturação das recomendações, sendo consideradas as publicações oficiais e científicas produzidas nos últimos 15 anos.

O processo de seleção e organização documental foi orientado por critérios de pertinência temática, credibilidade das fontes e atualidade das informações, assegurando a validade, consistência e representatividade do *corpus* analisado ^30^. Inicialmente, realizou-se a pré-análise e a categorização do material por meio de uma leitura exploratória e analítica, com o objetivo de identificar conteúdos relevantes, como recomendações, populações prioritárias e sustentação da proposta no contexto da natureza dos documentos (Quadro 1). Em seguida, procedeu-se à codificação sistemática das informações, visando construir um rol de estratégias por categorias interpretativas alinhadas a proposta de microeliminação das hepatites virais. Esse processo resultou na elaboração de um documento norteador com estratégias de enfrentamento.

**Quadro 1:** Principais referências documentais e material científico que subsidiaram a estruturação das recomendações de enfrentamento das hepatites virais para o grupo de trabalho. Região de Saúde Médio Norte Mato-grossense, Mato Grosso, Brasil.

REFERÊNCIA (ANO)	RECOMENDAÇÕES/POPULAÇÕES PRIORITÁRIAS/SUSTENTAÇÃO
Lazarus et al. ^47^ (2022)	Recomendação: definição clara da população ou território-alvo. Mapeamento da linha de base epidemiológica e de barreiras ao cuidado. Estratégias de testagem e tratamento adaptadas ao contexto. Uso de modelagem ou projeções para orientar metas locais. Envolvimento de governo, prestadores de serviços de saúde, sociedade civil e comunidades. Parcerias estratégicas (p.ex.: sistema prisional, programas de redução de danos, organizações não-governamentais - ONGs). Indicadores de testagem, início de tratamento e resposta virológica sustentada. Publicização de resultados e ajustes estratégicos periódicos. Uso de testes rápidos no ponto de cuidado e/ou coleta em locais comunitários. Estratégias de *reflex testing* (teste automático de RNA após sorologia positiva). Protocolos simplificados, inclusive com profissionais não especialistas. Integração com programas de HIV, serviços de saúde mental, centros de atenção a usuários de drogas, serviços prisionais e maternidades. Expansão do acesso em populações privadas de liberdade e regiões remotas. Modelos de *test-and-treat* com acompanhamento remoto. Estratégias específicas para populações com risco contínuo. Populações prioritárias: pacientes em hemodiálise ou com distúrbios hemorrágicos; pessoas vivendo com HIV; populações geograficamente delimitadas (municípios ou comunidades pequenas); população privada de liberdade (com protocolos bem estruturados). Sustentação: a microeliminação é mais efetiva quando combina planejamento estratégico, metas claras, coordenação multissetorial e monitoramento contínuo, com foco em populações específicas e ações integradas aos serviços já existentes.
Chen et al. ^51^ (2022)	Recomendação: triagem em massa e realizada em curto período (5 dias), abrangendo mais de 2/3 da população prisional. Testagem seguida de *reflex testing* (detecção imediata nos casos positivos). Acesso rápido ao tratamento. Populações prioritárias: para população privada de liberdade, tratamento imediato no local por meio de implementação de protocolo padronizado simplificado. Administração no próprio ambiente prisional, sem necessidade de encaminhamento externo. Aumenta adesão e reduz perdas por transferência ou liberação. Colaboração entre hepatologistas e autoridades prisionais. Excluir pacientes com liberação ou transferência previstas para garantir conclusão do tratamento. Acordos administrativos para evitar transferências durante o curso terapêutico. Integração com seguro público nacional, garantindo acesso universal à terapia. Sustentação: modelo baseado em triagem em massa, tratamento imediato, coordenação institucional sólida e fluxo terapêutico simplificado.
Corcoran et al. ^40^ (2023)	Recomendação: ampliação universal do acesso ao tratamento com indicação de tratamento para todas as pessoas. Substituição de protocolos complexos por simplificados, facilitando a prescrição em serviços de APS e contextos comunitários. Avaliação de fibrose por métodos não invasivos (p.ex.: FIB-4, elastografia). Fluxos reduzidos de consultas e exames. Uso de contatos remotos (telefone/telemedicina) para suporte e adesão. Cirrose descompensada, HCC e transplante hepático requerem encaminhamento especializado. Triagem e tratamento simultâneo de coinfecções HBV/HIV quando presentes. Integração na APS: qualificação de equipes não especialistas para prescrição e seguimento de DAAs. Telemedicina e dispensação ampliada: entrega de todo o esquema terapêutico no início do tratamento. Educação em saúde e adesão simplificada: foco em uso diário da medicação, sem monitoramento intensivo. Vigilância e prevenção de reinfecção: testagem anual para grupos com risco contínuo. Sustentação: modelo simplificado baseado nas diretrizes AASLD/IDSA para ampliar o acesso ao tratamento e logística simplificada capaz de implementar estratégias de microeliminação em diferentes populações e territórios, especialmente nos níveis locais e regionais da rede de saúde.
Lazarus et al. ^82^ (2018)	Recomendação: matriz de estratégias de microeliminação + populações-alvo: Planejamento focado em população bem definida. Estabelecer metas anuais ou de curto prazo de diagnóstico, tratamento e cura. Processo multissetorial/participação de *stakeholders*, envolvendo governo, serviços de saúde, sociedade civil, outras partes interessadas para implementar o plano. Acompanhar e publicar indicadores (prevalência, diagnóstico, tratamento, cura, mortes) de forma transparente. Adaptar a testagem, cuidado, tratamento conforme barreiras, cultura, localização da população-alvo. Em populações de alto risco de transmissão, tratar acelera redução da incidência (*treatment as prevention*). Em populações de alto risco de transmissão, há potencial para tratamento como prevenção, se se tratar um bom volume de indivíduos, reduz‐se a incidência. Sustentação: abordagem de microeliminação não substitui o plano nacional ou generalizado de eliminação: ela é um instrumento estratégico para focar esforços em populações ou territórios definidos. Necessidade de justificar por que certas populações são priorizadas, garantindo que nenhuma população seja ignorada indefinidamente (princípio de “realização progressiva” de direitos à saúde).
Departamento de HIV, Aids, Tuberculose, Hepatites Virais e Infecções Sexualmente Transmissíveis, Secretaria de Vigilância em Saúde e Ambiente, Ministério da Saúde ^44^ (2025)	Recomendação: descentralização do cuidado para a APS com ampliação do acesso ao diagnóstico, tratamento e seguimento clínico por equipes multiprofissionais. Microplanejamento territorial com a construção de planos locais de ação com diagnóstico situacional, definição de linhas de cuidado, metas e responsabilidades para rastreamento, tratamento e monitoramento. Busca ativa e testagem focalizada com priorização de grupos de maior vulnerabilidade para testagem rápida de HBV/HCV, diagnóstico precoce e vinculação ao cuidado Fortalecimento da cascata de cuidado: estratégias para garantir alta taxa de diagnóstico, tratamento e retenção em cuidado, com indicadores claros de impacto e processo. Ações integradas de prevenção: ampliação da cobertura vacinal para hepatite B, controle de biossegurança em procedimentos invasivos e redução de danos entre populações vulnerabilizadas. Participação social e combate ao estigma: engajamento de movimentos sociais, populações afetadas e organizações locais no desenho e execução das estratégias. Monitoramento e avaliação contínua: uso de sistemas de informação (SINAN, SIM, SI-PNI, Siclom) e metas pactuadas para medir avanços e corrigir rotas. Populações prioritárias: gays e outros HSH; pessoas trans e travestis; profissionais do sexo; pessoas em situação de rua; pessoas privadas de liberdade; pessoas que usam drogas injetáveis, inaláveis ou fumadas; alcoolistas. Grupos com histórico de exposição: pessoas que receberam transfusão antes de 1993; pessoas submetidas a procedimentos invasivos sem biossegurança adequada; pessoas em terapia renal substitutiva; profissionais de saúde e segurança expostos a material biológico. Pessoas com condições clínicas específicas: pessoas vivendo com HIV e/ou aids; pessoas coinfectadas HBV/HDV (especialmente na Amazônia Legal); pessoas transplantadas. Grupos populacionais específicos: população indígena, ribeirinha e quilombola; pessoas gestantes e crianças expostas ao HBV (com enfoque na prevenção da transmissão vertical). Sustentação: estratégias territorializadas, equitativas e mensuráveis, com foco na descentralização do cuidado, testagem focalizada, prevenção combinada e monitoramento contínuo.
Queiroz et al. ^52^ (2021)	Recomendação: realizar testagem rápida em todos os novos detentos, seguida de confirmação com RNA-HCV. Uso de terapias pangenotípicas para reduzir tempo até o início do tratamento e minimizar barreiras logísticas. Possibilidade de protocolos baseados apenas em exames laboratoriais quando o acesso à imagem for limitado. Coordenação entre secretarias de saúde e administrações prisionais para garantir infraestrutura mínima e continuidade do cuidado. Campanhas educativas para detentos, profissionais penitenciários e visitantes. Ações para reduzir compartilhamento de objetos cortantes, uso de drogas injetáveis e práticas de risco. Redução da superlotação e melhoria das condições sanitárias, com criação de espaços adequados para atendimento em saúde. Implementação de protocolos de acompanhamento dos casos e reinfecções. Utilização de dados do Departamento Penitenciário Nacional para microplanejamento. População prioritária: pessoa privada de liberdade. Sustentação: fortes barreiras estruturais e institucionais favorece a microeliminação da hepatite no sistema prisional por meio de estratégias coordenadas e governança intersetorial entre saúde e segurança pública
Huang et al. ^63^ (2023)	Recomendação: testagem aplicada em populações “fechadas” (hemodiálise, prisões, áreas hiperendêmicas) ou “abertas” (populações móveis, p.ex.: pessoas que usam drogas injetáveis, HSH, trabalhadoras do sexo, população trans). Estratégia *test-and-treat* com triagem ampliada e início rápido de DAAs. Testagem no local (*point of care*), telemedicina, equipes móveis e integração com serviços já existentes (HIV, PrEP, redução de danos). Vigilância contínua e prevenção da reinfecção, especialmente em grupos com risco contínuo. Recomendação de testagem HCV RNA a cada 3-6 meses em populações de risco. Envolvimento de diferentes setores (saúde, justiça, sociedade civil). Campanhas nacionais integradas com metas claras para cada subpopulação. Populações prioritárias: pacientes em hemodiálise; populações em áreas hiperendêmicas; pessoas privadas de liberdade; HSH, com ou sem HIV; pessoas trans e gênero-diversas; pessoas que usam drogas injetáveis; trabalhadores(as) do sexo. Sustentação: estratégias de microeliminação adaptadas ao contexto epidemiológico e social são fundamentais para atingir a eliminação. A integração com serviços já existentes e o monitoramento de reinfecções são componentes críticos para a sustentabilidade dos resultados.
Bajis et al. ^43^ (2020)	Recomendação: microeliminação tanto a subpopulações (p.ex.: pessoas privadas de liberdade, pessoas que usam drogas injetáveis) quanto a territórios (p.ex.: municípios ou regiões sanitárias). Definir metas anuais de testagem, tratamento e monitorar indicadores em tempo real para avaliar desempenho local e fazer ajustes rápidos. Uso de sistemas de vigilância e avaliação periódica de resultados. Testagem simplificada (testes rápidos, *reflex testing*), diagnóstico precoce e início imediato de DAAs. Tratamento em APS, unidades de redução de danos, prisões e centros comunitários. Uso de telemedicina, farmácias comunitárias e enfermagem ampliada para prescrição e seguimento. Participação ativa de populações-chave no desenho e implementação das estratégias. Ações de comunicação e advocacia para ampliar a adesão e reduzir barreiras socioculturais. Populações prioritárias: pessoas que usam drogas; pessoas privadas de liberdade; pacientes em diálise ou com distúrbios hemorrágicos; pessoas vivendo com HIV; migrantes e minorias étnicas; coortes de nascimento de alta prevalência; trabalhadores do sexo e pessoas trans; áreas geográficas hiperendêmicas/territórios prioritários com possibilidade de eliminação territorial acelerada com microplanejamento. Sustentação: microeliminação é uma estratégia essencial para acelerar o progresso rumo à eliminação da hepatite. Ela depende de focalização inteligente em populações específicas, uso de testagem simplificada e tratamento imediato, descentralização do cuidado, engajamento comunitário e monitoramento robusto.
Morales-Arraez et al. ^53^ (2021)	Recomendação: uso de telemedicina para prescrição, seguimento e apoio a pacientes, especialmente em locais de difícil acesso ou com barreiras estruturais. Descentralização do tratamento: dispensação de terapias de HCV fora dos centros especializados, aliando cuidado mais próximo do paciente e modelos de atenção comunitária ou situada. Modelo de cuidado que reengaja pacientes que estavam perdidos no seguimento ou não haviam acessado tratamento, aumentando a cobertura terapêutica, o que favorece a lógica de “tratamento como prevenção” (*treatment as prevention*) em microeliminação. Integração de tecnologia, sistemas de apoio remoto e coordenação entre níveis de atenção à saúde para facilitar acesso, adesão e monitoramento. Populações prioritárias: pacientes com HCV que vivem em áreas rurais ou remotas, com acesso limitado a hepatologistas ou serviços especializados, beneficiam-se especialmente da telemedicina + dispensação descentralizada. Grupos vulneráveis ou com barreiras de acesso ao cuidado tradicional (p.ex.: pessoas com mobilidade reduzida, populações marginalizadas, ou em regiões de baixa densidade de serviços), para os quais esse modelo representa uma “ponte”. Populações-chave para microeliminação, em que a ampliação rápida de tratamento pode gerar impacto em transmissão/incidência se combinada à cobertura adequada. Sustentação: combinação de telemedicina com tratamento descentralizado pode funcionar como uma estratégia catalisadora para a microeliminação, especialmente em contextos em que o acesso ao tratamento convencional é dificultado. Esse modelo permite ampliar cobertura terapêutica, reduzir barreiras logísticas e acelerar o impacto em populações-alvo.
Botto et al. ^59^ (2024)	Recomendação: testagem reflexa ou em etapa única (*reflex/single-step testing*) na APS, serviços hospitalares, programas de testagem em populações prioritárias. Uso do antígeno core (HCV cAg) como alternativa diagnóstica. Testes moleculares no ponto de cuidado (POC NAAT) como realização do teste confirmatório diretamente no local de atendimento (p.ex.: GeneXpert, Genedrive), incluindo coleta por punção digital. Populações prioritárias: pessoas que injetam drogas; pessoas privadas de liberdade; populações em situação de rua ou vulnerabilidade social; pacientes atendidos na atenção primária e emergências hospitalares; populações com alta prevalência local identificada por vigilância ou inquéritos sorológicos.; indivíduos com histórico de exposição a sangue (p.ex.: usuários de serviços de transfusão antes de 1993, pacientes em hemodiálise, etc.). Fortalecimento da rede comunitária para alcance das populações prioritárias. Utilização de plataformas móveis para ampliar cobertura em territórios vulneráveis. Ajustes regulatórios para permitir descentralização da confirmação diagnóstica. Monitoramento contínuo dos indicadores de testagem, confirmação, tratamento e cura para cada subpopulação-alvo. Sustentação: a estratégia mais custo-efetiva e com maior impacto na eliminação da hepatite C é a implementação de fluxos reflexos ou em etapa única combinados a abordagens POC e uso complementar de HCV cAg, especialmente quando direcionados a populações de maior vulnerabilidade epidemiológica.
Von den Hoff et al. ^41^ (2022)	Recomendação: descentralização total do cuidado em centros de atenção ao uso de substâncias com testagem, aconselhamento, prescrição de DAAs e acompanhamento clínico são realizados no próprio serviço de atenção à dependência. Integração entre equipes de saúde e rede de atenção à dependência com profissionais de saúde mental, enfermagem e médicos de adição atuam na triagem, prescrição e monitoramento e especialistas em hepatites dão suporte remoto e presencial para casos complexos. Uso de DAAs pangenotípicos e protocolos simplificados, pois elimina necessidade de genotipagem prévia e facilita início rápido do tratamento e adesão. A avaliação clínica não invasiva com uso de FIB-4 e Child-Pugh para estratificação de risco e, em casos sem cirrose ou cirrose compensada são tratados onsite; casos complexos são referenciados para especialistas. Modelo RE-AIM para implementação e avaliação (*Reach, Effectiveness, Adoption, Implementation, Maintenance*) com foco em ampliar cobertura, medir efetividade real, identificar barreiras e planejar expansão e ações de vinculação ativa ao cuidado com abordagem de *low-threshold care* (baixa exigência), focada em eliminar barreiras práticas (pouca burocracia, coleta no local, integração com tratamento da dependência química) e testagem e início do tratamento no mesmo local e momento sempre que possível. População prioritária: pessoas que usam drogas injetáveis. Sustentação: descentralização completa do cuidado para hepatite dentro dos serviços de atenção à dependência é efetiva e viável, alcançando maior cobertura e adesão. O modelo remove barreiras logísticas e clínicas tradicionais, permitindo tratamento no local e ampliando o alcance entre pessoas que usam drogas. Estratégia considerada replicável em outros contextos nacionais e internacionais, especialmente em regiões com alta prevalência concentrada nessa população.
Matthews et al. ^49^ (2023)	Recomendação: estratégias centrais para microeliminação da hepatite B com focalização em populações bem definidas ou regiões geográficas delimitadas. Aplicação da lógica de microeliminação: definição de subpopulações ou territórios específicos com metas claras de curto e médio prazo, articuladas com as metas nacionais de eliminação. Expansão da vacinação universal e estratégias complementares com garantia de cobertura vacinal de ≥ 95% em crianças (três doses) e dose ao nascer e vacinação de grupos de risco, incluindo contatos domiciliares, profissionais de saúde e populações vulneráveis. Além de ter, a vacinação como pilar de prevenção da transmissão vertical e horizontal. Implementação de testagem focalizada e *opt-out* em serviços de atenção primária, maternidades, prisões e locais de alta prevalência. Integração da testagem para HBV com programas já existentes (HIV, HCV, IST, saúde materno-infantil). Testagem universal de gestantes, profilaxia adequada e vacinação neonatal imediata. Inclusão da profilaxia com antivirais para gestantes HBsAg positivas com alta carga viral, conforme diretrizes internacionais. Identificação ativa de indivíduos com infecção crônica elegíveis para tratamento. Acompanhamento clínico descentralizado para os demais casos, com estratificação de risco (p.ex.: ALT, HBV DNA, FibroScan ou APRI/FIB-4). Microeliminação pode ser usada como abordagem piloto para populações de difícil acesso, informando políticas nacionais. Definição de indicadores mensuráveis (p.ex.: cobertura vacinal, testagem, tratamento, supressão viral, mortalidade). Uso de sistemas de informação integrados e vigilância epidemiológica ativa. Populações prioritárias: gestantes e crianças; profissionais de saúde; pessoas que usam drogas; pessoas privadas de liberdade; migrantes de países de alta endemicidade; populações indígenas e minorias étnicas; pessoas coinfectadas (HIV, HCV, HDV). Sustentação: microeliminação da hepatite B exige estratégias multifocais, com ênfase na prevenção da transmissão vertical, vacinação universal, diagnóstico ampliado e cuidado descentralizado em populações de maior vulnerabilidade.
Villa & Navas ^67^ (2023)	Recomendação: testagem para em 100% das gestantes, independentemente de fatores de risco. Base operacional da microeliminação por coorte materno-infantil. Profilaxia antiviral materna. Monitoramento e seguimento do binômio mãe-bebê com testagem sorológica do bebê aos 6-12 meses, registro nacional de mães positivas e acompanhamento de filhos para avaliar falhas vacinais, além de fortalece vigilância epidemiológica e rastreamento. Treinamento de profissionais de saúde em com identificação precoce de gestantes, aplicação oportuna de vacina e aconselhamento sobre amamentação segura (não contraindicada se mamas íntegras). Integração com programas de saúde reprodutiva e planejamento familiar. População prioritária: gestantes. Sustentação: microeliminação da hepatite B passa pela eliminação da transmissão vertical, eixo estruturante do controle global. Testagem universal, imunoprofilaxia combinada e antivirais no final da gestação são as ações mais custo-efetivas para romper o ciclo de infecção crônica.
Barreira et al. ^60^ (2024)	Recomendação: ampliação da testagem rápida e sorológica para populações e territórios de maior risco (zonas rurais, Amazônia, populações vulneráveis). Implantação de fluxos para diagnóstico molecular (HBV-DNA, HCV-RNA, HDV-RNA, HEV IgM) nos pontos de atenção estratégicos. Início precoce do tratamento antiviral (particularmente para HCV com DAAs), reduzindo tempo entre diagnóstico e terapia. Fortalecimento da cobertura vacinal contra hepatite B e imunização de contatos e grupos vulneráveis. Identificação proativa de casos agudos e contatos, inclusive em populações de difícil acesso. Integração com programas de HIV, tuberculose, IST e doenças crônicas para abordagem integral. Vigilância genômica para identificar introduções de novos genótipos e monitorar dispersão regional. Estratégias articuladas com atenção primária e redes regionais de saúde para garantir rastreio, encaminhamento e tratamento. Populações prioritárias: pessoas ≥ 20 anos não vacinadas ou com status vacinal desconhecido (HBV); pessoas > 40 anos; povos indígenas, quilombolas e ribeirinhos (Região Norte); filhos de mães HBsAg ou HCV positivas; pessoas em situação de rua; profissionais de saúde, estética e segurança pública; pessoas privadas de liberdade; pessoas que usam drogas (injetáveis, inaladas ou fumadas); HSH, travestis e transexuais; PVHA (pessoas vivendo com HIV/aids); pessoas em hemodiálise ou com doença renal crônica; gestantes e mulheres em planejamento reprodutivo; população geral em áreas endêmicas (Amazônia); Pessoas com histórico de transfusão pré-1993; pacientes com doenças hepáticas, ISTs e imunossuprimidos. Sustentação: intervenções rápidas em *clusters* com confirmação laboratorial, mapeamento de contatos e tratamento no território. Fortalecimento da logística de insumos e antivirais, garantindo acesso contínuo ao tratamento e a capacitação de equipes de saúde (inclusive APS) com integração com vigilância genômica e epidemiológica para identificação precoce de surtos ou introduções de genótipos emergentes.
Departamento de Vigilância, Prevenção e Controle das IST, do HIV/Aids e das Hepatites Virais, Secretaria de Vigilância em Saúde, Ministério da Saúde ^36^ (2018)	Recomendação: eixos estratégicos da microeliminação da hepatite C. Prevenção: comunicação e educação em saúde voltadas a grupos vulneráveis. Prevenção combinada (biomédica, comportamental e estrutural). Redução de danos e insumos preventivos para populações-chave. Orientação a contatos domiciliares e parceiros sexuais. Diagnóstico: ampliação do rastreio com teste rápido e biologia molecular (HCV-RNA). Testagem populacional em massa, principalmente > 40 anos. Diagnóstico dirigido a populações prioritárias. Mapeamento territorial de serviços e busca ativa de casos. Vigilância pós-diagnóstico para evitar perda de seguimento. Tratamento: tratamento universal com DAAs. Início rápido após diagnóstico, incluindo casos leves (F0-F3). Encaminhamento a centros especializados apenas para casos complexos (carcinoma hepatocelular , falha terapêutica, coinfecções graves). Gestão e linha de cuidado: integração da APS e serviços especializados com referência e contrarreferência. Notificação, vinculação e seguimento clínico organizado. Educação permanente e capacitação das equipes. Uso estratégico dos sistemas de informação para rastreabilidade. Simplificação do fluxo de acesso, com tratamento iniciado em serviços locais (APS/CTA/SAE). Populações prioritárias: Grupo prioritário 1 - testagem frequente (pelo menos 1x/ano ou trimestral em PrEP): pessoas vivendo com HIV/aids. Pessoas em uso de PrEP. Pessoas privadas de liberdade. Pessoas com múltiplos parceiros sexuais ou IST. Travestis, transexuais, gays e HSH. Trabalhadores(as) do sexo. Pessoas que usam álcool e outras drogas. Pessoas em terapia renal substitutiva Grupo prioritário 2 - testagem ao longo da vida: Pessoas ≥ 40 anos. Pessoas com diabetes. Antecedente psiquiátrico, doença renal ou imunossupressão. Histórico de transfusão de sangue ou hemoderivados antes de 1992. Exposição a material biológico contaminado. Uso de álcool ou drogas. Tatuagem/*piercing* em locais não regulamentados. Doença hepática sem diagnóstico, ALT/AST elevadas. Comunicantes e parceiros(as) de pessoas com HCV. Pessoas nascidas de mães HCV positivas. Sustentação: foco populacional capaz de priorizar grupos com maior prevalência e risco de transmissão. Foco territorial: ações dirigidas a regiões de maior carga de doença. Integração: APS, CTA/SAE, vigilância e gestão municipal/estadual. Meta OMS 2030: reduzir 90% das infecções e 65% da mortalidade por HCV.
Departamento de Vigilância, Prevenção e Controle das Infecções Sexualmente Transmissíveis, do HIV/Aids e das Hepatites Virais, Secretaria de Vigilância em Saúde, Ministério da Saúde ^45^ (2018)	Recomendação: ampliação do acesso com utilização de testes rápidos para triagem de hepatites B e C em unidades de APS, CTA/SAE, prisões, serviços móveis e populações-chave. Complementação diagnóstica com realização de testes moleculares (PCR em tempo real) para confirmação da infecção ativa (HBV-DNA, HCV-RNA). Algoritmos padronizados visto que o manual apresenta 8 fluxogramas diagnósticos para HAV, HBV, HCV, HDV e HEV, com destaque para fluxos 1 e 4 (testagem inicial HBV e HCV) e fluxos 2 e 5 (confirmação molecular). Notificação e sistemas com uso integrado do gerenciador de ambiente laboratorial (GAL) para rastreio laboratorial, SINAN para notificação e Sisloglab para insumos laboratoriais. Avaliação da qualidade com o Programa de Avaliação Externa da Qualidade para execução de testes rápidos. Locais estratégicos para testagem: unidades básicas de saúde (APS), CTA/SAE, Consultório na Rua, Rede Cegonha. serviços de emergência, hospitais, maternidades. Unidades móveis e ações itinerantes. Populações prioritárias: Hepatite B: HSH, profissionais do sexo, pessoas que usam drogas, pessoas privadas de liberdade, indivíduos em situação de rua, indígenas, quilombolas, indivíduos nascidos em áreas endêmicas; Hepatite C: indivíduos com 40 anos de idade ou mais, indivíduos que realizaram transfusão, transplante, indivíduos em situação de compartilhamento de material de injeção. Sustentação: reforça a integração com vigilância epidemiológica e bancos de dados nacionais. Uso intensivo de testes rápidos em cenários móveis e APS. Vigilância ativa em comunicantes e contatos, com priorização de gestantes, pessoas privadas de liberdade e populações rurais.
Grande et al. ^54^ (2021)	Recomendação: ampliação da vacinação contra HBV e HAV, incluindo esquemas combinados. Vacinação específica em populações vulneráveis (HSH, usuários de drogas, gestantes, imunossuprimidos). Medidas de higiene e saneamento para prevenção de HAV e HEV. Rastreamento em bancos de sangue para HEV e HBV. Testagem em populações de maior risco, incluindo testagem reflexa e pontos de cuidado (*point-of-care testing*). Teste e tratamento integrados, com início de terapias antivirais assim que o diagnóstico for confirmado. Protocolos simplificados para HCV com base em PCR/antígeno e sem necessidade de genotipagem extensa. Terapia universal com DAAs para HCV, com regimes pangenotípicos e curtos (8-12 semanas). Uso ampliado de análogos de nucleotídeos para HBV (entecavir, TDF, TAF), com perspectiva de novas terapias imunomoduladoras e antivirais experimentais (bulevirtide, VIR-2218, *capsid inhibitors*). Manejo de HEV em grupos de risco com ribavirina em casos selecionados. Descentralização para APS e serviços comunitários. Tratamento conduzido também por profissionais não especialistas (enfermeiros, médicos da APS, equipes prisionais). Integração vigilância-cuidado para rastrear casos não tratados. Identificação de indivíduos previamente diagnosticados e não tratados (HCV *lost to follow-up*). Estratégias de testagem ampliada em prisões, serviços de saúde mental, centros de uso de drogas e serviços de diálise. Uso de dados epidemiológicos locais para mapear *hotspots* de transmissão. Rastreamento em populações com baixa vinculação ao sistema de saúde (migrantes, indígenas, usuários de drogas, privados de liberdade). Populações prioritárias: indivíduos HIV-positivos que são suscetíveis à infecção por HAV; população psiquiátrica, pessoas com dependência de drogas ou em terapia de reposição, pacientes com insuficiência renal ou receptores de transplante de órgãos sólidos; Sustentação: ações direcionadas a grupos de maior risco, ampliação da testagem, tratamento universal e descentralização dos serviços. Disponibilidade de regimes terapêuticos curtos e altamente eficazes.
Song et al. ^37^ (2022)	Recomendação: fortalecimento da vigilância sentinela e notificação compulsória. Prevenção direcionada a grupos de maior risco (usuários de drogas, HSH, privados de liberdade). Prevenção de infecções hospitalares, protocolos rigorosos de biossegurança, esterilização e controle em hemodiálise. Implantação de triagem ampliada e proativa, com testagem reflexa (teste rápido → HCV-RNA) em grupos vulneráveis. Inclusão de anti-HCV em *check-ups*, pré-operatórios e pré-natal. Integração com APS e vigilância epidemiológica. Criação de redes integradas de prevenção e tratamento entre atenção primária, hospitais e Centros de Testagem locais. Modelos de gestão local para referência e contrarreferência ágil. Simplificação de fluxos de cuidado, testagem, diagnóstico e tratamento no mesmo serviço. Ampliação da cobertura de DAAs via negociações de preço e incorporação ao seguro de saúde nacional. Desenvolvimento de DAAs locais para reduzir custos e dependência de importações. Uso de regimes pangenotípicos simplificados (8-12 semanas). Campanhas de educação pública e treinamento profissional para aumentar a conscientização sobre HCV. Mobilização comunitária e parcerias locais para melhorar a adesão ao tratamento. Adoção de estratégias de microeliminação territorial e populacional, entrada por populações de alto risco e locais de maior prevalência. Populações prioritárias: pessoas que usam drogas (injeção ou inalável), HSH e pessoas com múltiplos parceiros sexuais. Pessoas privadas de liberdade, pessoas coinfectadas com HIV ou HBV, populações com práticas sexuais de risco, migrantes e populações vulneráveis com baixa cobertura de serviços. Sustentação: investimento no foco em grupos vulneráveis como porta de entrada para ampliação de cobertura, tendo integração entre a APS a vigilância e os centros especializados para diagnóstico e tratamento descentralizados. Negociação e produção local de DAAs para reduzir barreiras econômicas. *Test-and-treat* simplificado com fluxos rápidos e adesão monitorada. Microeliminação escalável: iniciar em subpopulações de alto risco e expandir para populações gerais.
Huang et al. ^42^ (2025)	Recomendação: testagem em massa com *reflex testing* (teste rápido + carga viral) para HCV. Rastreamento rotineiro integrado ao cuidado de HIV (testagem semestral ou anual). Ações focalizadas em HSH com risco elevado e pessoas em PrEP. Disponibilidade irrestrita de DAAs desde 2017. Início imediato do tratamento após confirmação da viremia. Regimes pangenotípicos e esquemas curtos (8-12 semanas; 6-8 semanas para infecção aguda). HCV incorporado no fluxo de cuidado de HIV: testagem, tratamento e monitoramento pós-RVS (pós-resposta virológica sustentada). Alta cobertura: > 99% dos pessoas vivendo com HIV testados, > 94% tratados. Testes de RNA ou antígeno HCV a cada 6 meses (EASL/OMS), com estratégias de testagem agrupada (*pooled RNA testing*) a cada 3 meses para alto risco. Identificação precoce de reinfecções e início imediato de DAA. Educação sobre práticas sexuais seguras. Integração com programas de PrEP e prevenção combinada. Campanhas comunitárias para reduzir estigma e ampliar acesso. População prioritária: HSH HIV-negativos. Sustentação: integração da testagem e tratamento de HCV ao cuidado de HIV é uma estratégia altamente eficaz. Programas bem-sucedidos devem incluir monitoramento intensivo da reinfecção, tratamento universal com DAA e intervenções comportamentais combinadas.
The Lancet HIV ^61^ (2018)	Recomendação: foco inicial em populações bem definidas permite resultados rápidos e eficientes. Diagnóstico universal em pessoas vivendo com HIV. Vinculação imediata aos serviços especializados e início de tratamento. Ações integradas com redes de HIV para rastrear e tratar coinfectados. Acesso garantido aos DAAs no sistema de saúde nacional. Eliminação de barreiras de elegibilidade para pacientes coinfectados. Estratégias para manejo da reinfecção. Foco em subpopulações de difícil acesso (pessoas em situação de rua, usuários de drogas injetáveis, minorias étnicas, pessoas com doença mental). Desenvolvimento de serviços móveis e *outreach* para rastreamento e tratamento. Estratégia integrada com políticas e programas de HIV. Testagem combinada e ações preventivas coordenadas. Necessidade de políticas claras para usuários em reinfecção e retratamento, bem como ampliação da força de trabalho para serviços de testagem. Populações prioritárais: Pessoas vivendo com HIV (particularmente HSH). Pessoas que usam drogas injetáveis. Pessoas privadas de liberdade. Pessoas em situação de rua. Indivíduos com transtornos mentais graves. Subpopulações vulneráveis: Minorias étnicas e imigrantes com baixa vinculação a serviços de saúde. Pessoas sem acesso a PrEP e redes estruturadas de HIV. Populações com baixa testagem. Sustentação: a microeliminação é apresentada como um catalisador para avançar em direção à eliminação nacional, mesmo diante de barreiras logísticas e políticas. Pode gerar efeito cascata com redução de novas infecções e maior engajamento de populações de difícil acesso. Permite ações direcionadas com impacto epidemiológico rápido, especialmente em redes de coinfecção HIV-HCV.
Hollande et al. ^57^ (2020)	Recomendação: aumento da cobertura de testagem com testes rápidos de diagnóstico (RDOTs) e testes *point-of-care*. Estratégias de rastreamento ampliadas para além dos grupos clássicos de risco. Identificação de indivíduos não diagnosticados e reengajamento de pacientes perdidos de seguimento. Transferência dos serviços diagnósticos e terapêuticos para fora dos hospitais, com forte participação da atenção primária e serviços móveis. Expansão do acesso aos DAAs em serviços comunitários. Formação de equipes multiprofissionais para rastrear e tratar populações vulneráveis. Eliminação de barreiras de elegibilidade e priorização do tratamento para todos os pacientes com viremia detectada. Regimes pangenotípicos curtos (8-12 semanas). Estratégia *test-and-treat* no mesmo dia. Monitoramento ativo da incidência e prevalência por subpopulação e território. Prevenção combinada com redução de danos, programas de troca de seringas e salas de consumo supervisionado. Integração com programas de HIV, hepatites B e ISTs. Início de programas com subpopulações bem definidas e de alta prevalência, facilitando o alcance de metas rápidas e mensuráveis. Populações prioritárias: pessoas que usam drogas injetáveis, pessoas privadas de liberdade, HSH, pessoas vivendo com HIV. Populações vulneráveis adicionais: migrantes oriundos de regiões endêmicas, pacientes em diálise e transplantados renais, pessoas com transtornos psiquiátricos graves, pessoas em situação de rua, populações complementares e clínicas, pacientes com cirrose e fibrose avançada, hemofílicos e pessoas que receberam transfusões antes da triagem sorológica, coortes etárias com alta prevalência (p.ex.: nascidos entre 1945-1965 em alguns países). Sustentação: focar em populações de alta prevalência permite obter impacto epidemiológico rápido e mensurável. Descentralizar o diagnóstico e o tratamento, e integrar HCV com outros programas de saúde. Políticas de prevenção combinada (redução de danos, testagem regular, tratamento universal) reduzem significativamente a incidência e a reinfecção.
Organização Pan-Americana da Saúde ^68^ (2025)	Recomendação: vacinar ≥ 95% dos bebês com 3 doses contra hepatite B, garantindo a primeira dose nas primeiras 24 horas de vida. Oferecer profilaxia antiviral com tenofovir para gestantes elegíveis. Reforçar esquemas de vacinação em grupos de risco e adultos suscetíveis. Oferecer triagem gratuita e acessível para HBV e HCV, com foco nos grupos de maior risco e integração de autotestes. Triagem universal de sangue doado e práticas seguras de injeção. Ampliação da cobertura de testagem em atenção primária e centros comunitários. Tratar 100% das pessoas diagnosticadas com HCV, utilizando regimes pangenotípicos altamente eficazes. Garantir 80% de cobertura de tratamento para pessoas com HBV elegíveis. Oferecer testagem e tratamento próximos ao local de moradia, APS, centros comunitários, prisões e clínicas móveis. Fortalecer redes de referência e contrarreferência. Monitoramento contínuo de pessoas com sequelas de hepatites B e C, com rastreamento para carcinoma hepatocelular e acompanhamento clínico. Práticas de injeção e transfusão 100% seguras. Fortalecimento da vigilância de eventos adversos e monitoramento laboratorial. Sustentação: a estratégia da Organização Pan-Americana da Saúde (OPAS) para hepatites B e C baseia-se em prevenção da transmissão vertical, triagem universal, tratamento universal com regimes pangenotípicos, descentralização dos serviços, e foco em populações prioritárias.

APS: atenção primária à saúde; AASLD/IDSA: American Association for the Study of Liver Diseases/Infectious Diseases Society of America; CTA/SAE: Centro de Testagem e Aconselhamento/Serviço de Assistência Especializada; DAA: antivirais de ação direta; EASL/OMS: European Association for the Study of the Liver/Organização Mundial da Saúde; HAV: vírus da hepatite A; HBsHg: antígeno de superfície do vírus da hepatite B; HBV: vírus da hepatite B; HCC: hepatcarcinoma; HCV: vírus da hepatite C; HDV: vírus da hepatite D; HEV: vírus da hepatite E; HSH: homens que fazem sexo com homens; IST: infecções sexualmente transmissíveis; PrEP: profilaxia pré-exposição; SI-PNI: Sistema de Informação do Programa Nacional de Imunizações; Siclom: Sistema de Controle Logístico de Medicamentos; SIM: Sistema de Informações sobre Mortalidade; SINAN: Sistema de Informação de Agravos de Notificação; Sisloglab: Sistema de Controle Logístico de Insumos Laboratoriais.

Fonte: elaboração própria a partir da revisão narrativa.

Diante da ausência de uma análise diagnóstica da gestão do cuidado integral em perspectiva regional, o grupo gestor organizou, no segundo momento, um grupo focal. Os achados da análise documental e bibliográfica foram essenciais para a elaboração do roteiro dos grupos focais, definindo eixos temáticos de discussão e orientando a construção coletiva de consensos na etapa seguinte da pesquisa. Foram convidados para o grupo focal, via e-mail, o gestor estadual da área técnica de hepatites virais, o gestor regional de hepatites virais do Escritório Regional de Saúde, coordenadores municipais de hepatites virais, de APS e de vigilância epidemiológica dos dez municípios da RS, dois enfermeiros gestores de unidades da APS e gestores do serviço de referência para hepatites virais. A seleção, por conveniência, deve-se ao enfoque da pesquisa e à possibilidade de reunir *stakeholders* em um único momento. Os critérios de inclusão foram: vínculo mínimo de seis meses ao cargo, atuação como articuladores da área de hepatites virais nos municípios da RS, disponibilidade para a reunião e aceite em participar da pesquisa.

Os dados do segundo momento foram coletados em junho de 2023, por meio de três grupos focais, sendo dois com enfermeiros da APS e um com gestores municipais e do serviço de referência. Cada grupo contou com moderador, co-moderador e dois observadores responsáveis pela gravação, controle do tempo e registro em diário de campo ^31^. Utilizou-se roteiro elaborado pelos pesquisadores, previamente validado (face e conteúdo) e pré-testado, que contemplava perfil socioeconômico e profissional, além de questões sobre abordagem às hepatites virais na unidade de saúde, ações voltadas à população-alvo, práticas de gestão e regulação assistencial, dificuldades, monitoramento de casos e da rede de serviços. Os encontros, audiogravados e com duração média de quatro horas, tiveram as anotações de campo integradas aos dados da pesquisa.

O material foi transcrito na íntegra e submetido à análise temática, escolhida por sua capacidade de identificar, analisar e relatar padrões (temas) nos dados, organizando-os de forma detalhada e oferecendo uma abordagem teórica flexível e útil para gerar relatos ricos e complexos ^32^. O *corpus* da análise foi então relacionado à matriz de gestão do cuidado integral em rede regionalizada de saúde, com foco na integração e regulação assistencial ^32^.

A matriz de análise fundamenta-se no referencial teórico-metodológico da gestão do cuidado integral, estruturada em três níveis interdependentes: político-institucional, organizacional e das práticas ^33^. 

No nível político-institucional, inclui estratégias de expansão e fortalecimento da rede, adequação estrutural das unidades, definição do escopo da APS e regulação para referência, contrarreferência e acompanhamento longitudinal. No nível organizacional, abrange pactuação de metas regionais, conectividade das unidades, incentivos e apoio institucional, incorporação tecnológica, integração entre APS e serviços especializados e mecanismos que reforcem o papel coordenador da APS. Já no nível das práticas, considera a cobertura e completude das equipes de saúde da família, critérios de encaminhamento, acompanhamento contínuo dos casos, uso de tecnologias de apoio, comunicação entre profissionais e usuários e reconhecimento da APS como referência habitual ^33^. Esse arranjo analítico permite compreender como diretrizes, arranjos institucionais e práticas cotidianas se articulam na construção de redes regionalizadas e integradas.

Aplicada a atenção às hepatites virais, a matriz permite avaliar em que medida os níveis se articulam para garantir acesso, continuidade e integralidade. Os pressupostos analíticos foram construídos a partir da capacidade da RAS na RS em formular e pactuar estratégias de enfrentamento no âmbito político-institucional; organizar fluxos, integrar APS e serviços especializados, incorporar tecnologias e sustentar mecanismos de regulação no nível organizacional; e assegurar, no cotidiano das equipes, acompanhamento longitudinal, comunicação efetiva e práticas que reduzam desigualdades no nível das práticas. Dessa forma, a matriz possibilita analisar como a regulação assistencial orienta caminhos para fortalecer a governança regional e a microeliminação desse agravo.

No terceiro momento, em junho de 2023, durante o I Simpósio Regional de Enfrentamento das Hepatites Virais da Região Médio Norte de Mato Grosso, profissionais e gestores da RS envolvidos na atenção às hepatites virais participaram de grupos focais. O grupo gestor articulou os dados em contraste com o documento norteador produzido na etapa documental e bibliográfica, sintetizados nos Quadros 1 e 2, resultando em uma apresentação dos principais pontos a serem enfrentados.

Ao final do encontro, os participantes da plenária realizaram a leitura prévia do documento norteador para subsidiar a elaboração do Plano de Ação para o Enfrentamento das Hepatites Virais na Região Médio Norte Mato-grossense, 2023-2030. Cada linha estratégica foi discutida e ajustada conforme as manifestações dos participantes, sob condução de um coordenador, dois relatores e quatro apoiadores responsáveis pela gravação, controle do tempo e registro das sínteses. Ressalta-se que o plano resulta da integração ensino-serviço-gestão, articulando ensino, pesquisa e extensão na experiência colaborativa do Escritório de Qualidade para Organizações de Saúde (EsQualOS) com a gestão municipal e regional ^34^, derivado de ações subsequentes à publicação de pesquisa ^35^.

Quanto aos aspectos éticos, todos os participantes assinaram o Termo de Consentimento Livre Esclarecido, aceitaram ter suas falas gravadas nos grupos focais e durante a plenária no evento, autorizaram que o material fosse utilizado para divulgação científica, sendo que esse produto é fruto de pesquisa aprovada (Escola de Enfermagem de Ribeirão Preto, Universidade de São Paulo; CAAE: 01481918.0.0000.5393).

**Quadro 2:** Governança da rede na perspectiva de atores sobre os espaços decisórios no processo regional. Região de Saúde Médio Norte Mato-grossense, Mato Grosso, Brasil, 2023.

GOVERNANÇA DA REDE	ANÁLISE DA PRÁTICA DE GOVERNANÇA	PRINCIPAIS EXTRATOS DE FALAS DOS PARTICIPANTES
Coordenação Estadual da área de hepatites	Relato de dificuldades na regulação assistencial para casos de alta complexidade como hepatocarcinomas e ausência de fluxo formal Defende-se a contrarreferência estruturada	“*Hoje, nós não temos um fluxo estabelecido que parta do CTA para nenhum ponto de intervenção em alta complexidade. Não há encaminhamento claro para transplante, por exemplo. Fica tudo dependendo de articulações pontuais, o que é muito frágil diante da gravidade desses casos*” “*Essa contrarreferência vai precisar ser montada com base em protocolos bem definidos. O paciente precisa ter um cartão, um instrumento de registro e identificação, para que possamos rastrear o seu percurso e garantir que ele não fique perdido na rede*”
Coordenação regional da área de hepatites	Apoio técnico contínuo oferecido aos municípios, especialmente no que se refere à interpretação dos resultados e orientação clínica Reconhece limitações estruturais importantes, como a falta de informatização nos serviços	“*A gente está o tempo todo apoiando os municípios. Tem muita dúvida, eu mesmo,* [risos]*, vivo com dúvidas sobre como interpretar os marcadores da hepatite. Aí eu ligo, pra RT ou para a farmacêutica e elas me explicam, orienta passo a passo*” “*É um apoio muito direto, porque sabemos que há dificuldade mesmo, principalmente onde a rotatividade é alta ou a capacitação não chega com qualidade*” “*Elas estão fazendo tudo sem nenhum tipo de sistema informatizado. Tudo no papel. E aí, como é que acompanham a trajetória desse paciente? Como saber se ele já fez o exame, se voltou pra consulta, se iniciou tratamento? Fica tudo muito solto, e isso prejudica o cuidado*”
Secretários Municipais de Saúde	Em alguns municípios, há relatos de ausência de coordenação. Diferenças significativas na estruturação da resposta. Em alguns municípios, a fragilidade institucional compromete diretamente a execução das ações O engajamento da gestão facilita a implementação, especialmente o apoio direto do secretário de saúde, é um fator decisivo para a efetividade da resposta	“*A maior dificuldade lá é essa: não tem coordenação, não tem uma equipe de vigilância epidemiológica estruturada, sem um responsável que puxe o processo ou organize os fluxos. Isso atrasa tudo e fragiliza as ações*” “*Quando a gestão está engajada, tudo anda com mais facilidade. Fazer acontecer é mais viável quando o secretário de saúde está junto, apoiando, acompanhando de perto. Isso faz toda a diferença para que a equipe se sinta respaldada e as ações saiam do papel*”
Comissão Intergestores Regional (CIR)	Não há menções diretas à atuação da CIR nas falas transcritas, sugerindo uma ausência de articulação regionalizada via CIR em relação às hepatites	Não houve registro
Conselhos Municipais de Saúde	Citado como instância de apoio em situações específicas. Não há menções de atuação contínua ou estruturada	“*A gente até aciona o Conselho, chama uma vez, chama duas, chama três, mas nem sempre há retorno ou participação efetiva. Eles aparecem quando a situação aperta, mas não têm uma presença constante ou articulada com as ações de planejamento e acompanhamento da rede*”

CTA: Centro de Testagem e Aconselhamento; RT: responsável técnico.

Fonte: elaboração própria.

## Resultados

A apresentação dos resultados foi organizada a partir do *framework* teórico-metodológico ^33^ adotado neste estudo, que concebe a gestão do cuidado integral em redes regionalizadas em três dimensões analíticas, político-institucional, organizacional e das práticas.

A análise documental permitiu sintetizar, no Quadro 3, as evidências sobre a articulação intersetorial na RAS na RS. Verificou-se que a maioria dos municípios apresenta alta cobertura da APS e depende da rede privada para exames laboratoriais e atenção especializada, enquanto, no caso das hepatites virais, as ações de testagem, diagnóstico e tratamento são realizadas integralmente no SUS.

**Quadro 3:** Características do sistema de saúde da Região de Saúde (RS) que abordam atenção às hepatites. Região de Saúde Médio Norte Mato-grossense, Mato Grosso, Brasil, 2023.

Divisão político-administrativa	Possui dez municípios (1. Tangará da Serra, 2. Arenápolis, 3. Barra do Bugres, 4. Campo Novo do Parecis, 5. Denise, 6. Nova Marilândia, 7. Nova Olímpia, 8. Porto Estrela, 9. Santo Afonso, 10. Sapezal)
Características da RS	População de 253.894 habitantes, apenas um município possui mais de 100 mil habitantes e quatro possuem menos que 10 mil. A economia concentra-se em parte considerável do agronegócio
Características de serviços na RS	13 postos de saúde, 65 centros de saúde/unidade de saúde, 19 policlínicas, 2 unidades mistas, 9 hospitais, 15 unidades de atenção à saúde indígena, 3 prontos atendimentos, 3 centros de atenção a hemoterapia e/ou hematologia, 3 laboratórios de saúde pública, 9 centrais de regulação do acesso. A cobertura de APS é de 90,07%. No serviço ambulatorial e hospitalar prevalece a rede privada
Referência para atenção às hepatites	Prevenção e testagem - unidades de APS nos municípios Diagnóstico e tratamento - centralizado no CTA/SAE da RS
Incidência média anual e prevalência acumulada de casos de hepatites B e C na RS	Hepatite B Casos totais (2018-2023): 5 Incidência média anual: 0,33 por 100.000 habitantes Prevalência acumulada (2018-2023): 1,97 por 100.000 habitantes Hepatite C Casos totais (2018-2023): 28 Incidência média anual: 1,84 por 100.000 habitantes Prevalência acumulada (2018-2023): 11,03 por 100.000 habitantes Hepatite B Casos totais (2024-2025): 0 Hepatite C Casos totais (2024-2025): 7 Incidência média anual: 1,38 por 100.000 habitantes Prevalência acumulada (2024-2025): 2,76 por 100.000 habitantes Interrupção de tratamento hepatite B Casos totais (2021-2023): 1

APS: atenção primária à saúde; CTA/SAE: Centro de Testagem e Aconselhamento/Serviço de Assistência Especializada;

Fonte: dados extraídos do Instituto Brasileiro de Geografia e Estatística ^83^, Departamento de Informação e Informática do SUS ^84^ e Ministério da Saúde ^85^ ^,^ ^86^ ^,^ ^87^.

A análise diagnóstica a partir de fontes secundárias subsidiou a seleção de 53 profissionais, *stakeholders* da rede de atenção da RS para as hepatites virais, dos quais 45 participaram do estudo, incluindo 20 gestores. Entre os gestores, 18 (90%) eram mulheres, 15 (75%) autodeclarados brancos, 16 (80%) tinham entre 30 e 40 anos, 14 (70%) eram casados, todos possuíam vínculo estatutário (100%) e mais de quatro anos de formação (100%).

Destacou-se a presença de coordenadores da APS, gestores de serviços ambulatoriais, de pronto atendimento, de laboratórios públicos e da rede hospitalar, além de responsáveis pela vigilância epidemiológica, pelo serviço de referência (CTA/SAE), pela coordenação estadual do programa de hepatites virais e pelo responsável técnico do Escritório Regional de Saúde. No espaço da gestão, registrou-se a ausência do Conselho Estadual de Secretários Municipais de Saúde (COSEMS) e de representantes dos Conselhos Municipais de Saúde. Entre os 25 enfermeiros da APS participantes, 21 (84%) eram mulheres, 12 (48%) pardos, 12 (48%) tinham entre 30 e 40 anos, 15 (60%) eram casados, 20 (80%) estatutários, 20 (80%) com mais de cinco anos de formados, 3 (25%) possuíam mestrado, apenas 2 (8%) tinham especialização em APS e 15 (60%) atuavam há mais de quatro anos na APS.

O Quadro 2 aborda a governança da rede sob a ótica dos atores envolvidos na gestão e nos espaços decisórios. Há consenso sobre a efetividade do CTA/SAE, referência regional para as hepatites virais, apontado como principal articulador no território, sendo inclusive um centralizador da expertise na RS. Destaca-se a formação técnica e a estabilidade da equipe, majoritariamente composta por servidores públicos, o que assegura continuidade das ações e legitimidade nos espaços deliberativos e na articulação intersetorial. A Comissão Intergestores Regional (CIR), coordenada pelo Escritório Regional de Saúde, consolidou-se como instância decisória com ampla participação dos municípios. Contudo, historicamente, ações específicas para hepatives virais foram pouco debatidas em reuniões ou assembleias deliberativas como a CIR, o COSEMS e a Comissão Intergestores Bipartite (CIB), configurando restrição de um dos espaços de governança, situação que começou a mudar a partir de 2021, com orientações do Ministério da Saúde. Não houve menção à participação dos Conselhos Municipais de Saúde. Reconhece-se o protagonismo da APS na prevenção, embora ainda haja desafios para incorporar as diretrizes atuais de testagem e tratamento no âmbito do SUS.

O Quadro 4, mediante a análise bibliográfica, sintetiza os achados sobre a integração e regulação assistencial na RS. No nível político-institucional, observa-se a ausência de estudos sobre vazios assistenciais e o baixo investimento específico para hepatites virais, com centralidade da resposta concentrada no SAE. Não foram registradas barreiras ao acesso ao diagnóstico ou ao tratamento com apoio do Serviço de Apoio Diagnóstico e Terapêutico (SADT). No nível organizacional, predomina o atendimento pelo SUS, com o setor privado atuando apenas na oferta de exames nos municípios sem estrutura laboratorial pública. O SAE realiza os exames confirmatórios dos casos encaminhados. A testagem se concentra na APS, mas também ocorre, pontualmente, em pronto atendimentos e laboratórios. Os registros ainda são manuais, e a telessaúde é pouco utilizada, com exceção do SAE. No nível das práticas, há definição de fluxos de referência que orientam a atuação dos profissionais; entretanto, a ausência de sistemas informatizados prejudica o acompanhamento dos usuários referenciados. Dúvidas clínicas são resolvidas de forma informal, via telefone ou aplicativos de mensagens, evidenciando a necessidade de protocolos clínicos e capacitação específica para o manejo das hepatites virais.

**Quadro 4:** Análise multinível da atenção às hepatites virais por meio das dimensões político-institucionais, organizacionais e de práticas na regulação assistencial, seus desafios e potencialidades na perceptiva da governança da regionalização. Região de Saúde Médio Norte Mato-grossense, Mato Grosso, Brasil, 2023.

NÍVEIS	PRINCIPAIS EXTRATOS DE FALAS DOS PARTICIPANTES	DESAFIOS IDENTIFICADOS	POTENCIALIDADES AFIRMADAS
Nível político-institucional			
Não há registros de estudos ou estratégias voltadas à identificação e superação de vazios assistenciais	“*Sinceramente, nunca tivemos um levantamento claro sobre os vazios assistenciais na nossa região. A gente sabe, no dia a dia, que tem municípios com pouca estrutura, mas nunca foi feito um estudo que mostre isso com dados, nem uma estratégia concreta para resolver. Parece que vamos tapando buracos conforme os problemas aparecem, mas sem um planejamento mais estruturado*”	Ausência de estudos/estratégias para identificação de vazios assistenciais Baixo investimento específico em hepatites Centralização da regulação no SAE Falta de informatização e prontuário eletrônico Protocolos clínicos pouco adaptados às realidades locais	Rede de serviços é majoritariamente pública Inexistência de filas de espera permite resposta rápida após o diagnóstico Acesso garantido aos SADT
Apesar da rede de serviços para hepatites ser majoritariamente pública, o investimento específico nessa área permanece reduzido	“*A rede de serviços para hepatites é pública, isso é claro, todo mundo sabe. Mas, na prática, o investimento específico nessa área é muito pequeno*”		
A marcação de exames e consultas para hepatites concentra-se no SAE, com pouca atuação da central de regulação	“*A marcação de exames e consultas para hepatites aqui funciona mais pela gestão direta do SAE. É algo que ficou meio institucionalizado com o tempo, talvez pela agilidade ou pela proximidade com os casos*”		
Não houve menção ao uso de prontuários eletrônicos na gestão dos casos de hepatite	“*O mais irônico é que, em plena era digital, a gente ainda trabalha tudo no papel. São mais de 2.000 prontuários manuais. Não temos um sistema, nada informatizado. Isso dificulta muito o acompanhamento longitudinal dos casos, e torna o processo lento e sujeito a perdas. Sem prontuário eletrônico, a gestão do cuidado fica desconectada*”		
Os protocolos clínicos utilizados baseiam-se quase exclusivamente em documentos do Ministério da Saúde, com pouca adaptação às realidades locais	“*A gente segue os protocolos do Ministério da Saúde, é o que temos. Mas não tem uma adaptação local, sabe? Cada território tem suas especificidades, mas ninguém parou para discutir ou construir algo mais próximo da nossa realidade. Então, usamos o que está disponível, mesmo que nem sempre se encaixe direito no nosso contexto*”		
Não há filas de espera para tratamento, o que permite resposta rápida após o diagnóstico, possivelmente devido ao baixo número de casos notificados	“*Hoje, não temos fila de espera para tratamento. Os casos são poucos e, quando aparece um diagnóstico, a resposta é quase imediata. A oferta de tratamento acontece de forma rápida, justamente porque o volume de notificações é muito baixo. É uma vantagem, mas também nos faz pensar se estamos realmente conseguindo alcançar todos que precisam*”		
Não há registros de restrições ao acesso aos SADT	“*Sobre exames mais complexos, felizmente não enfrentamos grandes barreiras. Até onde sabemos, não há restrição no acesso ao SADT. Quando é necessário, conseguimos encaminhar. Mas claro, isso pode mudar de região para região, e às vezes depende muito da articulação local*”		
Nível organizacional			
Constata que o acesso às hepatites é totalmente público no contexto estudado	“*O acesso das hepatites é todo feito pelo SUS, não existe serviço privado envolvido nesse fluxo. No entanto, falta uma integração entre os serviços. Cada setor parece agir de forma isolada, sem diálogo horizontal. A gente sente que falta um sistema que articule a rede como um todo, algo que facilite a vida de quem precisa percorrer esse caminho até o tratamento*”	Falta de integração horizontal entre os serviços Centralização excessiva das referências no SAE Testagem desigual e pulverizada entre municípios Laboratórios municipais sem estrutura para confirmação diagnóstica Concentração de ações ambulatoriais no município sede Regulação manual, sem informatização Ausência de protocolos regionais Ausência de telessaúde Problemas de logística no transporte sanitário	Atendimento integralmente público (via SUS) Organização de mutirões pela APS em municípios menores Oferta de SADT disponível, ainda que com falhas operacionais
Não foi observada integração horizontal entre os serviços; há centralização das referências, mas sem ordenamento articulado do apoio diagnóstico	“*Mesmo quando existe uma referência definida, ela não vem acompanhada de um ordenamento dos serviços de apoio diagnóstico. Isso dificulta muito o trabalho em rede e a lógica de cuidado compartilhado*”		
A realização dos testes está centralizada na APS em alguns municípios, enquanto em outros é pulverizada entre ambulatórios, pronto atendimento e APS	“*A testagem acontece de formas bem diferentes nos municípios. Em alguns lugares, é a atenção básica que assume tudo; em outros, os testes aparecem só em mutirões ou em ambulatórios. Cada município foi se organizando do seu jeito, mas isso gera desigualdades e confusão sobre onde procurar o serviço*”		
A incorporação tecnológica é restrita ao serviço de referência; laboratórios municipais não possuem equipamentos para confirmação diagnóstica	“*Nos municípios menores, os laboratórios públicos não têm estrutura para confirmação diagnóstica. Eles só conseguem fazer a testagem inicial. Tudo que envolve confirmação tem que ser enviado ao serviço de referência. Isso atrasa o processo e depende muito do fluxo funcionar bem*”		
As ações ambulatoriais concentram-se no município sede; os demais organizam mutirões pela APS	“*As consultas e acompanhamentos acontecem praticamente só no município sede, onde está o serviço de referência. Os demais municípios acabam fazendo mesmo é os mutirões e ações pontuais organizadas pela APS. Isso mostra uma concentração do cuidado, sem um fluxo contínuo nos territórios menores*”		
Não foi mencionada informatização da regulação para consultas e exames	“*A gente ainda marca exame e consulta tudo de forma manual ou por telefone. Não há informatização no processo de regulação. Isso gera atraso, pode ser que haja possíveis erros de comunicação. Nem falo em retrabalho, porque isso é sim, sempre*”		
Não há instrumentos formais de referência e contrarreferência	“*Ainda não chegamos a dialogar sobre instrumentos formais de referência e contrarreferência. Quando o paciente vai e volta do serviço de referência, não há registro estruturado, nem retorno oficial, o acompanhamento do caso é lá mesmo no serviço de referência, acho que o paciente até prefere*”		
Não há uso de prontuário eletrônico; protocolos são internos ao serviço de referência	“*Não temos prontuário eletrônico, e cada profissional faz seus registros no físico ainda. Os protocolos usados foram desenvolvidos internamente pelo serviço de referência, mas não há padronização, é mais restrito a eles*”		
O recurso da telessaúde não é utilizado pelos municípios como suporte às hepatites	“*A telessaúde, infelizmente, não é utilizada, seria uma ferramenta valiosa, mas não, não é nossa realidade*”		
Há oferta de SADT, mas ocorrem falhas de transporte por desencontros de agendas	“*Em termos de exames, temos acesso aos serviços de apoio diagnóstico. O problema maior tem sido a logística do transporte. Às vezes, a agenda da regulação não bate com a agenda do serviço, e o paciente perde a viagem. Mas não é por falta de transporte. Os municípios costumam reagendar para garantir o atendimento*”		
Nível de práticas			
O usuário transita entre diferentes pontos da rede pública, mas sem garantia de continuidade integrada do cuidado	“O *usuário circula entre diferentes pontos da rede, mas a comunicação do problema dele, isso depende muito da iniciativa dos profissionais. Em teoria, há uma rede funcionando. Na prática, é como se fosse uma trilha com partes desconectadas, onde cada serviço atua isoladamente, sem um sistema que garanta a continuidade do cuidado*”	Rede fragmentada, sem continuidade integrada do cuidado Falta de prontuário eletrônico para acompanhamento Comunicação entre serviços de forma informal e sem institucionalização Ausência de protocolos municipais próprios Monitoramento concentrado no SAE, sem contrarreferência à APS	Oferta de vagas suficiente para atender à demanda atual Contatos informais entre APS e SAE funcionam como mecanismo ágil para resolver demandas pontuais
A oferta de vagas tem atendido à demanda, embora possa refletir subnotificação de casos	“*Em relação às consultas e procedimentos, não temos enfrentado fila de espera. A quantidade de vagas tem dado conta da demanda atual. Mas isso não quer dizer que o acesso está universalizado, pode ser também reflexo de subnotificação ou da dificuldade que o paciente enfrenta para percorrer o caminho completo do cuidado*”		
Há mecanismos de referência da APS, mas a ausência de prontuário eletrônico fragiliza a integração dos serviços	“*Os encaminhamentos da atenção básica para o serviço especializado existem, mas sem prontuário eletrônico. O histórico do paciente se perde entre os papéis. Não há como acompanhar de forma integrada. Muitas vezes, quando o paciente retorna para a unidade, a equipe sequer sabe em que etapa do cuidado ele está*”		
A comunicação entre profissionais e o serviço de referência ocorre de forma improvisada, via telefone	“*Hoje, o que acontece é que os profissionais da atenção básica ligam direto pro CTA/SAE. Ligam pra tirar dúvidas, entender resultado de exame, ou até pra tentar adiantar uma consulta. É um contato informal, que resolve no improviso, mas que também mostra a ausência de um canal oficial de comunicação entre os serviços*”		
Os municípios não possuem protocolos próprios, restringindo-se aos do serviço de referência	“*A gente até tenta seguir alguma linha, mas falta um documento orientador local, que traduza as diretrizes para a realidade dos territórios*”		
O acompanhamento dos usuários fica concentrado no serviço de referência, sem contrarreferência efetiva à APS	“*Depois que o paciente é diagnosticado, o acompanhamento fica totalmente sob responsabilidade do serviço de referência. Se ele não entra em tratamento medicamentoso, acaba se perdendo da rede. A atenção básica não retoma esse paciente, ele vira um ponto cego no cuidado. E isso acontece com frequência*” “*A contrarreferência praticamente não existe. O paciente vai ao CTA/SAE e, muitas vezes, não volta mais para a unidade de origem. Ele acha que todo o cuidado vai acontecer lá e não há um movimento claro de devolutiva. Com isso, a atenção básica perde completamente o vínculo e o acompanhamento*”		

APS: atenção primária à saúde; CTA: Centro de Testagem e Aconselhamento; SADT: Serviços de Apoio Diagnóstico e Terapêutico; SAE: Serviço de Assistência Especializada; SUS: Sistema Único de Saúde.

Fonte: elaboração própria, com base no banco de dados.

O Quadro 5 apresenta o Plano de Ação para o Enfrentamento das Hepatites Virais na Região Médio Norte Mato-grossense, 2023-2030, que define e prioriza ações e investimentos para incluir o tema na agenda estratégica do planejamento regional e local. 

**Quadro 5:** Plano de ação para o enfrentamento das hepatites virais na Região Médio Norte Mato-grossense, 2023-2030 definido por linhas estratégicas e estratégias para fomentar articulação regional na proposta de eliminação das hepatites virais. Região Médio Norte Mato-grossense, Mato Grosso, Brasil, 2023.

LINHA ESTRATÉGICA 1 - PROMOVER UMA RESPOSTA ABRANGENTE E INTEGRADA COM O ACESSO EQUITATIVO À ATENÇÃO PREVENTIVA
Estratégia 1 - Aumentar a descoberta de casos realizando exames de triagem: sorologia convencional ou teste rápido com enfoque na priorização das populações com maior risco de hepatites virais População-chave: 40 anos ou mais de idade - *baby Boomers*; Transfusão de sangue ou derivados antes de 1993; Uso de drogas injetáveis ou inaláveis; Diagnóstico atual ou anterior de IST; População prioritária inclusive os povos indígenas; Profissionais que manipulam perfuro cortante.
Estratégia 2 - Instituir/ampliar os exames de triagem nas unidades básicas de saúde e ambulatórios de especialidades Implementar a testagem rápida na rede de saúde, preferencialmente na APS; Implementar a testagem rápida na rede de pronto atendimento, para as situações de acidente ocupacional e violência sexual; Incentivar a Comissão de Controle de Infecção Hospitalar (CCIH) a ampliação da testagem relacionada a sinais e sintomas característicos das hepatites virais, a partir de alterações das enzimas transaminase oxalacética (TGO) e transaminase pirúvica (TGP); Ampliar a testagem rápida a pacientes diabéticos, hipertensos e com outras doenças crônicas.
Estratégia 3 - Testar todos os pacientes em acompanhamento nos serviços de ISTs/aids
Estratégia 4 - Prevenir a transmissão das hepatites virais B e C da mãe para o filho (transmissão vertical) Pesquisar o marcador HBsAg para diagnosticar hepatite B, tanto na testagem rápida, quanto na metodologia laboratorial realizado na rotina do pré-natal para todas as gestantes, no primeiro e terceiro trimestre; Pesquisar o marcador anti-HCV para triagem de hepatite C, tanto na testagem rápida, quanto na metodologia laboratorial - realizado no pré-natal para todas as gestantes, no primeiro e terceiro trimestre; Aplicar a vacina de hepatite B nas gestantes suscetíveis (anti-HBs não reagente) ainda não vacinadas ou com esquema vacinal incompleto; Tratar as gestantes com hepatite B com alta replicação viral (HBeAg reagente); Notificar todas as gestantes com suspeita de diagnóstico de hepatites B e C em até 7 (sete) dias; Gestante com HBsAg reagente ou anti-HCV reagente devem ser encaminhadas ao CTA/SAE imediatamente com resultados dos exames TGO, TGP e HBeAg; Todas as parcerias sexuais da gestante devem ser convocadas para testagem; Aplicar a primeira dose de vacina de hepatite B ao nascer (todos os recém-nascidos); Aplicar a imunoglobulina hiperimune contra hepatite B (HBIG) no pós-parto imediato para as crianças nascidas de mães portadoras de hepatite B; APS deverá acompanhar as crianças nascidas de mães portadoras de hepatite B, até o sexto mês de vida, e C, até 1 (um) ano de vida; Realizar contrarreferência, da maternidade para a APS, das crianças nascidas de mães portadoras de hepatites virais.
Estratégia 5 - Garantir transfusão de sangue e hemodiálise segura Realizar busca-ativa de usuários com marcadores para HBV e para HCV nos bancos de sangue; Realizar a investigação de casos de soroconversão de hepatites B e C em bancos de sangue - retrovigilância realizada conjuntamente pela vigilância epidemiológica e sanitária; Verificar se todos os pacientes com doença renal crônica encaminhados ao centro de hemodiálise realizam a testagem anti-HCV e HBsAg na abordagem inicial, e repetição da testagem de acordo com o protocolo.
Estratégia 6 - Garantir a ampliação da vacinação contra as hepatites A e B Acompanhar os esquemas básicos de vacinação infantil; Rastrear a população adulta que não foi vacinada contra a hepatite B; Manter e ampliar programas de imunização contra HBV a fim de aumentar a cobertura para todas as crianças, populações-chave e grupos vulneráveis; Garantir a vacinação de hepatite A à população específica, de acordo com critérios do Centro de Referência de Imunobiológicos Especiais (CRIE).
Estratégia 7 - Fortalecer a divulgação de informações à sociedade Implementar atividades de informação e comunicação e campanhas nos âmbitos regional e local para aumentar a conscientização sobre a existência, a gravidade e as vias de transmissão das hepatites virais e sobre as medidas para prevenir e controlar a doença; Aperfeiçoar a prevenção no território, com ampliação da educação em saúde sobre o agravo, em diferentes grupos sociais, com intervenções que podem ser guiadas por enfermeiros, inclusive com ações em ambientes escolares.
LINHA ESTRATÉGICA 2 - FOMENTAR O ACESSO EQUITATIVO AOS SERVIÇOS DE APS COM SUPORTE DE ATENÇÃO CLÍNICA ESPECIALIZADA E INFORMAÇÕES ESTRATÉGICAS
Estratégia 1 - Tratar os portadores de hepatites B e C de acordo com os protocolos de tratamento adotados no Brasil Ampliar o número de serviços para o atendimento de pacientes com hepatites B e C; Tratar todos os pacientes com hepatites B e C; Fornecer gratuitamente os medicamentos para tratamento das hepatites B e C; Implementar nos serviços de saúde o monitoramento dos pacientes com diagnóstico de hepatites B e C com retenção do paciente para a adesão ao tratamento; Criar um instrumento de contrarreferência para a unidade de saúde; Criar um cartão de controle para o paciente; Criar uma Unidade Dispensadora de Medicamentos (UDM) de acordo com a necessidade para a dispensação dos medicamentos do tratamento de hepatites B e C pela assistência farmacêutica do município; Trabalhar em parceria com a sociedade civil e sociedades médicas.
LINHA ESTRATÉGICA 3 - FORTALECER A CAPACIDADE LABORATORIAL PARA POSSIBILITAR O DIAGNÓSTICO
Estratégia 1 - Realizar os exames de biologia médica Analisar a capacidade de ampliação de unidades de coleta nos municípios da Região de Saúde.
LINHA ESTRATÉGICA 4 - FORTALECER A VIGILÂNCIA EM SAÚDE POR MEIO DE COMPROMISSOS PACTUADOS
Estratégia 1 - Pactuação da gestão estadual/regional Fortalecer a capacidade do setor da saúde de elaborar e implementar políticas e estratégias para prevenir a transmissão das hepatites virais entre populações-chave; Promover o desenvolvimento e a implementação de políticas e intervenções de saúde pública coordenadas a fim de erradicar as hepatites B e C; Promover a integração da prevenção, vigilância, diagnóstico, atenção e intervenções e serviços de controle para as hepatites virais no setor da saúde e implementá-los de forma coordenada e eficaz com os parceiros e as partes interessadas pertinentes; Promover a capacidade do setor da saúde de realizar as ações necessárias para promover a aplicação mais estrita de normas, protocolos e recomendações para prevenir a transmissão das hepatites virais em ambientes de atenção à saúde; Trabalhar na melhoria das informações com os municípios referente ao banco de dados: Sistema Nacional de Agravos de Notificação (SINAN), criança exposta ao HBV e HCV, gestantes, planilha de acompanhamento dos pacientes com hepatites B e C dos serviços municipais e publicar semestralmente a realização de reunião técnica sobre a situação de saúde das hepatites virais na região de saúde; Dar publicidade aos dados do boletim epidemiológico de hepatites virais anualmente; Realizar uma reunião a cada semestre com a Regional de Saúde para avaliação das ações necessárias em cada município; Propor em conjunto com a região de saúde a avaliação das ações necessárias para o alcance das ações pactuadas; Promover maior cooperação ao nível estadual/regional entre os programas de imunização, e hepatites virais, para integrar sistematicamente a ação estadual relacionada aos agravos no sistema estadual de saúde; Propor rede de serviço para todos os serviços de atenção terciária, em específico, a atenção oncológica (hepática); Fomentar, na região de saúde, a realização de ações em prol da eliminação das hepatites virais até 2030, na rede pública e privada; Garantir a realização de encontro de atualização/capacitação anualmente.
Estratégia 2 - Pactuação da gestão municipal e seus serviços de saúde Nomear responsável técnico para a área de hepatites no município; Os municípios deverão contemplar no Plano Anual de Saúde planejamento com prioridade das atividades para a eliminação das hepatites virais, a partir do ano de 2024; Implantar/fortalecer normas e padrões para a prevenção, diagnóstico, tratamento e monitoramento das hepatites virais; Estimular maior participação e integração das entidades da sociedade civil e grupos representantes na formulação de estratégias que visam acelerar a adoção da testagem, tratamento e demanda de outros serviços relacionados às hepatites virais; Estimular a obrigatoriedade da notificação compulsória das hepatites virais, identificando e sanando dificuldades, se necessário; Publicar anualmente a cobertura vacinal para hepatite A e B da população do território; Elaborar e implementar o fluxo da linha de cuidado das hepatites virais em seu território de acordo com as normatizações ministeriais, a partir do ano de 2024; Instituir anualmente, para equipe multiprofissional, capacitação para testagem e aconselhamento; Criar estratégias interprofissionais de atuação no território para prevenção e diagnóstico; Implantar normatização local para utilização de teste rápido na consulta de enfermagem, de acordo com as características clínicas e populações-chave para hepatites; Promover orientações para trabalhadores que se expõem ao risco da infecção das hepatites virais.
Estratégia 3 - Pactuação do serviço de referência regional Implementar fluxo assistencial e padrões para o rastreamento, diagnóstico, atenção e o tratamento das hepatites virais; Criar padrões para a referência e contrarreferência das hepatites virais; Fortalecer a atuação interprofissional para a atenção à saúde no serviço de referência, com apoio aos municípios para implementar o acompanhamento na rede local de serviços; Compartilhar conhecimentos e experiências com as equipes de saúde sobre o processo de trabalho na atenção com as pessoas com hepatites virais; Realizar/apoiar anualmente capacitações para os municípios da região de saúde.
Estratégia 4 - Pactuação com a instituição de Ensino Superior Realizar avaliações epidemiológicas das hepatites virais e de tecnologia em saúde; Pesquisar, analisar, publicar e difundir dados regionais sobre hepatites virais e sobre o impacto das respostas; Apoiar eventos científicos em parceria com o serviço especializado da região de saúde; Apoiar ações de extensão e cultura em parceria com os serviços municipais; Contribuir para a elaboração de políticas públicas de saúde às iniciativas de eliminação das hepatites virais; Pesquisar os recursos humanos disponíveis que são capazes de apoiar a eliminação da hepatite nos municípios; Desenvolvimento de uma base de evidências para o planejamento estratégico em prevenção e controle das hepatites virais; Apoiar a criação de tecnologias digitais da informação e comunicação (TDICs) em parcerias com os municípios para divulgação de informações sobre hepatites virais.

APS: atenção primária à saúde; CTA/SAE: Centro de Testagem e Aconselhamento/Serviço de Assistência Especializada; HbeHg: marcador da hepatite B; HBsHg: antígeno de superfície do vírus da hepatite B; HCV: vírus da hepatite C; IST: infecções sexualmente transmissíveis; PrEP: profilaxia pré-exposição.

Fonte: relatório final da Plenária do I Simpósio Regional de Enfrentamento das Hepatites Virais: Região Médio Norte Mato-grossense ^18^.

O plano fundamenta-se na lógica da RAS tendo a APS como ordenadora do cuidado e corresponsável pela atenção às hepatites virais. Embora elaborado a partir das especificidades regionais, mantém forte convergência com diretrizes nacionais e internacionais nos eixos da microeliminação das hepatites virais. Em agosto de 2023, foi submetido à CIR pelo Escritório Regional de Saúde, e apresentado aos dez secretários municipais, sendo homologado em setembro de 2023 pela *Resolução CIR Médio Norte nº 015, de 19 de setembro de 2023*. O documento contempla quatro linhas estratégicas principais.

(1) Promover uma resposta abrangente e integrada com o acesso equitativo à atenção preventiva: Trata de estratégias para ampliar a testagem, com foco nas populações-chave, organizar a triagem nos diferentes níveis de atenção, recomendar testagem em serviços de referência para infecções sexualmente transmissíveis (ISTs), prevenir a transmissão vertical, em transfusões de sangue e na hemodiálise, além de ampliar a vacinação contra hepatites A e B e fortalecer a disseminação de informações à sociedade.

(2) Fomentar o acesso equitativo aos serviços de APS com suporte de atenção clínica especializada e informações estratégicas: ratifica a necessidade de ampliar os serviços de atendimento às hepatites, reduzindo a centralização e promovendo acesso equitativo. O fortalecimento da atenção especializada, com dispensação monitorada de medicamentos, busca maior adesão ao tratamento e melhor controle no sistema de saúde. 

(3) Fortalecer a capacidade laboratorial para possibilitar o diagnóstico: expõe a necessidade de analisar a viabilidade de ampliar unidades de coleta nos municípios da RS, visando maior capilaridade da rede e redução do tempo de espera para exames confirmatórios.

(4) Fortalecer a vigilância em saúde por meio de compromissos pactuados: Apresenta a integração entre gestão, serviços e formação de recursos humanos em saúde para fortalecer a corresponsabilização por meio da pactuação de ações, definindo compromissos da gestão estadual/regional, municipal, do serviço de referência e da instituição de Ensino Superior.

O conjunto de resultados apresentados permite que os gestores municipais e regionais promovam amplo debate para o planejamento das ações subsidiados na perspectiva dos profissionais da rede de serviços de saúde, que em última análise podem ser considerados aqueles com estreita interface com os usuários. 

## Discussão

O estudo abordou três domínios interconectados, político-institucional, organizacional e das práticas, reconhecendo que as fragilidades na atenção às hepatites virais decorrem de interações complexas entre esses níveis. Uma resposta abrangente e equitativa exige que os *stakeholders* tenham clareza sobre as estratégias de microeliminação ^9^ ^,^ ^36^ ^,^ ^37^ e, no contexto do SUS, dada sua capilaridade e os condicionantes demográficos e socioeconômicos do acesso, demanda planos de trabalho alinhados à avaliação e ao monitoramento das ações traçadas ^25^.

Vale destacar que o nível político-institucional constitui o alicerce das respostas do sistema de saúde, exigindo investimento em dispositivos que fortaleçam a governança. Fragilidades nesse nível repercutem de forma ampla, comprometendo intervenções nos níveis organizacional e das práticas ^33^ ^,^ ^36^ ^,^ ^37^ ^,^ ^38^ ^,^ ^39^.

Os resultados revelam ausência de estratégias para identificar vazios assistenciais e fragilidades na organização da rede de referência, indicando lacunas na articulação intersetorial. Apesar da fluidez no início do tratamento e da ausência de barreiras ao SADT, o baixo investimento em informatização e gestão de dados evidencia a necessidade urgente de fortalecer a governança regional ^38^ ^,^ ^40^ ^,^ ^41^ ^,^ ^42^. Embora as hepatites virais tenham ganhado visibilidade na agenda nacional, sua efetivação é prejudicada por estruturas institucionais frágeis e dificuldades de articulação de grupos de *advocacy* ^11^ ^,^ ^43^. Essa limitação pode ser interpretada como reflexo da centralização da *expertise* em instâncias técnico-burocráticas restringindo a pluralidade de vozes, o que compromete a efetividade das respostas às hepatites virais em perspectiva da justiça social ^10^ ^,^ ^43^ ^,^ ^44^. 

Persistem lacunas na formulação de políticas baseadas em evidências e na participação efetiva dos *stakeholders*, sendo necessário tornar o tema transversal ao sistema de saúde no planejamento, com propostas sensíveis às singularidades locais e maior potencial resolutivo ^45^ ^,^ ^46^. É respeitável pontuar que qualquer avanço nas ações de enfrentamento das hepatites virais precisará conceber articulação intersetorial em redes integradas de serviços de saúde, mas também reconhecer que superar desafios e limites nas atividades intersetoriais solicitará engajamento articulado por meio da governança da sociedade civil, do setor saúde, das universidades, dos Conselhos de Saúde, das organizações não-governamentais e dos usuários. 

A regulação assistencial regional mostra-se complexa, marcada por desigualdades e entraves operacionais que limitam a implementação das propostas, apesar de sua relevância institucional e alinhamento nacional. Esse cenário, agravado por mudanças de governo, exige vigilância constante e análise que contemple dimensões técnicas, políticas, institucionais e culturais.

Na região estudada, o acesso organiza-se à margem do fluxo formal de regulação, centralizado no SAE, o que demonstra a necessidade de revisitar a regulação assistencial. A ausência de liderança municipal fragiliza a articulação intersetorial e compromete a responsabilização na execução dos planos ^36^ ^,^ ^40^ ^,^ ^44^ ^,^ ^47^ ^,^ ^48^. Evidenciaram-se fragilidades operacionais pelo uso de prontuários manuais e pela ausência de adaptação dos protocolos do Ministério da Saúde, o que centraliza informações e inviabiliza a gestão do cuidado em rede. O baixo investimento em sistemas de informação compromete o monitoramento, a avaliação e a comunicação entre serviços, refletindo também limitações político-institucionais decorrentes do financiamento de programas verticais ^36^ ^,^ ^44^ ^,^ ^49^ ^,^ ^50^.

No nível organizacional, foi notável a falta de promoção de uma articulação intersetorial e de integração horizontal dos serviços, o que reforça a constatação de que os serviços operam de forma isolada, inclusive pelas dificuldades de sincronização logística das consultas com o transporte. 

A limitação tecnológica da rede sobrecarrega o centro especializado, enquanto a capacidade de suporte clínico, que poderia fortalecer a resolutividade da APS e reduzir deslocamentos desnecessários por meio da telessaúde, foi pouco utilizada. A testagem nos municípios ocorre de forma pontual, sem diretriz regional ou coordenação pela APS, exceto na atenção à gestante. Investigações baseadas em trajetórias assistenciais revelam múltiplos pontos de abandono, decorrentes da falta de coordenação entre os serviços de saúde ^47^ ^,^ ^48^ ^,^ ^51^ ^,^ ^52^ ^,^ ^53^. A ampliação da testagem e garantia do tratamento exige fortalecimento da capacidade operacional dos laboratórios, com investimentos financeiros em regiões estratégicas, considerando aspectos epidemiológicos, demográficos e a adequada vinculação de recursos humanos, em quantidade e qualificação, para suprir a demanda e operar a tecnologia ^45^ ^,^ ^53^ ^,^ ^54^ ^,^ ^55^.

No nível de práticas, embora haja acesso a diferentes pontos da rede na RS, a articulação prática é insuficiente, não garantindo completude no atendimento visto as limitações de comunicação na rede. A suficiência de vagas favorece fluidez no encaminhamento, mas a centralidade do CTA/SAE pode restringir rastreio apenas à demanda espontânea, resultando em baixas taxas de diagnóstico e elevada subnotificação ^56^.

A microeliminação na literatura é justificada pela maior prevalência de hepatites virais em grupos como *baby boomers*, geração de pessoas nascidas no período pós-Segunda Guerra Mundial, geralmente entre 1946 e 1964, (cinco vezes mais risco de hepatite C) ^57^ ^,^ ^58^, pessoas que injetam drogas (prevalência de 44,82% para hepatite C e 2,66% para hepatite B) ^36^ ^,^ ^37^ ^,^ ^41^ ^,^ ^43^ ^,^ ^44^ ^,^ ^45^ ^,^ ^47^ ^,^ ^49^ ^,^ ^52^ ^,^ ^54^ ^,^ ^57^ ^,^ ^59^ ^,^ ^60^ ^,^ ^61^ ^,^ ^62^ e homens com parceiros sexuais masculinos, vulneráveis também à hepatite A ^36^ ^,^ ^37^ ^,^ ^42^ ^,^ ^44^ ^,^ ^63^ ^,^ ^64^. Ainda se exige sensibilização para prevenção em salões de beleza ^65^ e intensificação da testagem molecular em pacientes em hemodiálise ^47^ ^,^ ^60^ ^,^ ^66^, além da avaliação da rede municipal para prevenção da transmissão vertical ^41^ ^,^ ^44^ ^,^ ^67^ ^,^ ^68^ ^,^ ^69^. A prevalência entre populações quilombolas, indígenas e ribeirinhas ^49^ ^,^ ^52^ ^,^ ^54^ ^,^ ^60^ ^,^ ^70^ exige estratégias culturalmente adequadas e monitoramento que favoreça adesão ao tratamento.

A descentralização da testagem e dispensação de medicamentos, iniciada por diretrizes ministeriais ^71^ ^,^ ^72^, fortalece o acesso e amplia a corresponsabilidade por meio da governança regional. Expandir a cultura de triagem em múltiplos níveis da rede favorece diagnóstico e eliminação das hepatites virais ^37^ ^,^ ^41^ ^,^ ^43^ ^,^ ^44^ ^,^ ^46^ ^,^ ^47^ ^,^ ^49^ ^,^ ^51^ ^,^ ^53^ ^,^ ^57^ ^,^ ^59^ ^,^ ^68^. O uso de Sistemas de Apoio à Decisão Clínica, baseado em Registro Eletrônico de Saúde, é recomendado para aumentar a solicitação de testes de hepatite C, visto que informações específicas de cada pessoa, filtradas de forma inteligente, podem fornecer conhecimento às equipes de saúde em momentos apropriados ^53^ ^,^ ^73^.

É necessário reconhecer que o cuidado, sob a perspectiva da integralidade, está sustentado por interdependências entre os diferentes pontos da rede de atenção e na articulação intersetorial ^52^ ^,^ ^74^. Por isso, as fragilidades identificadas no estudo, especialmente nos processos de encaminhamento, na comunicação com outros pontos da rede e na construção de projetos terapêuticos para casos de maior complexidade clínica, evidenciam limitações importantes. Soma-se a isso o estigma social relacionado às hepatites virais, que leva muitos usuários a buscarem atendimento fora de seu município de origem para preservar o anonimato, dificultando ainda mais a articulação em rede ^56^ ^,^ ^75^.

Os resultados revelam uma tensão entre o papel atribuído ao CTA/SAE como matriciador e as barreiras estruturais (como a centralização dos serviços) e operacionais (problemas na comunicação interinstitucional e na fluidez dos encaminhamentos). Tais barreiras limitam sua capacidade de articular a rede de atenção regional, visto a demanda de atenção estar concentrada na *expertise* no CTA/SAE, que, embora valiosa, pode coexistir com gargalos assistenciais e acentuar iniquidades no acesso ao cuidado especializado, visto a centralidade da atenção ao único serviço ^48^ ^,^ ^76^.

Em análise da atenção às hepatites virais no Estado de Mato Grosso, a dimensão do acesso, no que tange à análise econômica, técnico-assistencial, política e simbólica, fica perceptível investimentos em políticas públicas que assegurem diagnóstico e tratamento, promovam organização do sistema na RAS em perspectiva integrada e sistêmica, que ampliem corresponsabilidade e reduzam estigmas, sobretudo com formação profissional apoiada na descentralização da atenção ^35^.

O estudo fortalece a perspectiva que, na gestão em saúde, a regulação do acesso deve ser compreendida como macrofunção estratégica, responsável por ordenar fluxos, garantir integralidade e reduzir desigualdades regionais. Os achados reforçam que fortalecer o acesso por meio de sua regulação implica reconhecer que seu fortalecimento não depende apenas de dispositivos técnicos, mas também de arranjos políticos e institucionais que sustentem a governança regional e ampliem a participação dos diferentes atores no processo decisório.

É crucial ampliar a divulgação sobre sintomas e serviços de atenção às hepatites virais ^35^. Experiências internacionais incluem ferramentas educativas no Japão, como materiais em áudio e o sistema de busca *Hepatic Navi* ^77^; na Índia, *webinars* ampliaram conhecimento ^78^; e na China ^79^, reestruturações na governança otimizaram recursos para ampliar acesso ao tratamento, respeitando as manifestações culturais e desigualdades territoriais, inserindo ações de gestão clínica, especialmente para população que apresenta alto risco para a infecção. Estratégias que demonstram oportunidade de parcerias com universidades para fomentar a disseminação de informação.

Ampliar a governança para a eliminação das hepatites virais é uma recomendação estratégica para o SUS, visto o seu modelo de gestão e a capilaridade de escuta que possui com a sociedade poderá construir pontes significativas de ações locais, regionais e estaduais, considerando as diferentes manifestações culturais e desigualdades no território brasileiro. 

No SUS a publicação do Programa Brasil Saudável projetou as hepatites virais em agenda prioritária das doenças socialmente determinadas, cujo enfrentamento exige mais do que respostas biomédicas, pois requer a articulação entre saúde, proteção social, saneamento, educação e participação comunitária ^10^. Nessa perspectiva, a gestão do cuidado como tecnologia social e o trabalho interprofissional configuram-se como dispositivos estratégicos para reorganizar modelos assistenciais e de governança para promover arranjos colaborativos capazes de integrar um projeto coletivo orientado pela equidade, pela justiça social e pela inovação institucional, reforçando a saúde como direito e prática civilizatória ^37^ ^,^ ^47^ ^,^ ^53^ ^,^ ^80^.

No que tange à proposta do plano elaborado, percebe-se que ela está alinhada às estratégias destacadas pelos gestores para avançar no enfrentamento das hepatites virais em Mato Grosso, as quais se concentram na ampliação do acesso, na integração da rede e no fortalecimento da capacidade técnica dos serviços. Entre as principais medidas, ressaltam-se: o reconhecimento e abordagem ativa de grupos prioritários no território com ampliação de parcerias intersetoriais; o monitoramento da transmissão vertical no pré-natal e parto; a integração entre áreas técnicas para descentralizar exames e organizar fluxos assistenciais; a expansão da testagem, especialmente na atenção primária, com estímulo à implantação de CTA em municípios que não dispõem desses serviços; a descentralização do tratamento com suporte de especialistas; a adoção de modelos organizacionais inspirados no cuidado ao HIV, visando desburocratizar o acesso; o uso estratégico das reuniões da CIR para sensibilização e pactuação de ações; e a capacitação contínua dos profissionais de saúde por meio da educação permanente ^81^. Essas estratégias convergem para uma resposta coordenada, territorializada e centrada no usuário.

Vale ressaltar que o avanço nessa construção coletiva é fruto da integração ensino-serviço-gestão que potencializa a resposta ao sistema de saúde e retoma a importância da articulação interinstitucional e na corresponsabilidade na formação de recursos humanos em saúde. Trata-se de valorizar a indissociabilidade universitária como estratégia para responder desafios complexos. Cria-se diretrizes estratégicas que podem ser monitoradas e avaliadas no intuito de garantir planejamento nos municípios, uma vez que foi consensuada em pactuação da CIR. Deverá ser ampla e transparente a divulgação do monitoramento, envolvendo dados sobre a situação de saúde e a implementações de ações políticas para a redução do agravo nos municípios e na RS. Pontua-se a proposta no âmbito do planejamento, o que vale então manter a atenção para que possa ser ampliada as ações propostas e revistas as estratégias, no caso das que não apresentarão resolutividade.

## Considerações finais

A análise evidenciou alta cobertura da APS na região, mas com forte dependência do setor privado para exames, enquanto as ações de testagem, diagnóstico e tratamento das hepatites virais concentram-se no SUS, configurando um modelo fragmentado e pouco articulado intersetorialmente. A centralidade do CTA/SAE como articulador regional e a frágil participação social nos espaços decisórios revelam um modelo de governança concentrado na *expertise* técnico-institucional. Além disso, lacunas em regulação, informatização e protocolos clínicos mantêm a rede excessivamente dependente de um serviço de referência específico. À luz dos pressupostos avaliativos adotados, tais achados confirmam que a governança regional carece de maior integração entre níveis político-institucional, organizacional e de práticas para sustentar a microeliminação das hepatites virais.

Tal cenário apresenta a necessidade de fortalecer a articulação política, padronizar condutas e investir em tecnologia e informação, elementos essenciais para avançar na eliminação das hepatites virais. Avanços como a definição de fluxos de referência e a formulação de um plano de ação regional para 2023-2030 incorpora o planejamento, acompanhamento e possibilidades de melhoria das políticas voltadas às hepatites virais.

Os resultados apontam a necessidade de parcerias sólidas entre profissionais, gestores, usuários, sociedade civil e governos. A microeliminação das hepatites virais requer não só avanços clínicos e tecnológicos, mas também a capacidade do sistema de atuar de forma colaborativa, intersetorial e territorializada, com espaços contínuos de pactuação e corresponsabilização. Nesse sentido, a regulação assistencial deve ser entendida como processo técnico e político, marcado pela coordenação de interesses e pela construção de compromissos coletivos com a equidade e a integralidade do cuidado.

Entre as limitações, destaca-se o fato de o estudo não ter incorporado a perspectiva dos usuários e da participação social, atores que poderiam oferecer interpretações distintas e complementares às dos gestores e profissionais da rede. A adoção de um estudo qualitativo pode restringir a complexidade do fenômeno em análise da rede. Contudo, a participação de profissionais diretamente envolvidos na gestão do sistema e na condução dos serviços fortalece a validade analítica dos resultados, confere densidade interpretativa e maior proximidade com os processos decisórios institucionais.

## Data Availability

As fontes de informação utilizadas no estudo estão indicadas no corpo do artigo.
